# Water Quality Monitoring with the Multiplexed Assay MitoOxTox for Mitochondrial Toxicity, Oxidative Stress Response, and Cytotoxicity in AREc32 Cells

**DOI:** 10.1021/acs.est.3c09844

**Published:** 2024-03-19

**Authors:** Jungeun Lee, Maria König, Georg Braun, Beate I. Escher

**Affiliations:** † Department of Cell Toxicology, UFZ—Helmholtz Centre for Environmental Research, 04318 Leipzig, Germany; ‡ Environmental Toxicology, Department of Geosciences, Eberhard Karls University, Schnarrenbergstr. 94-96, 72076 Tübingen, Germany

**Keywords:** mitochondrial membrane potential, mitochondrial toxicity, oxidative stress, environmental monitoring, mixture, AREc32

## Abstract

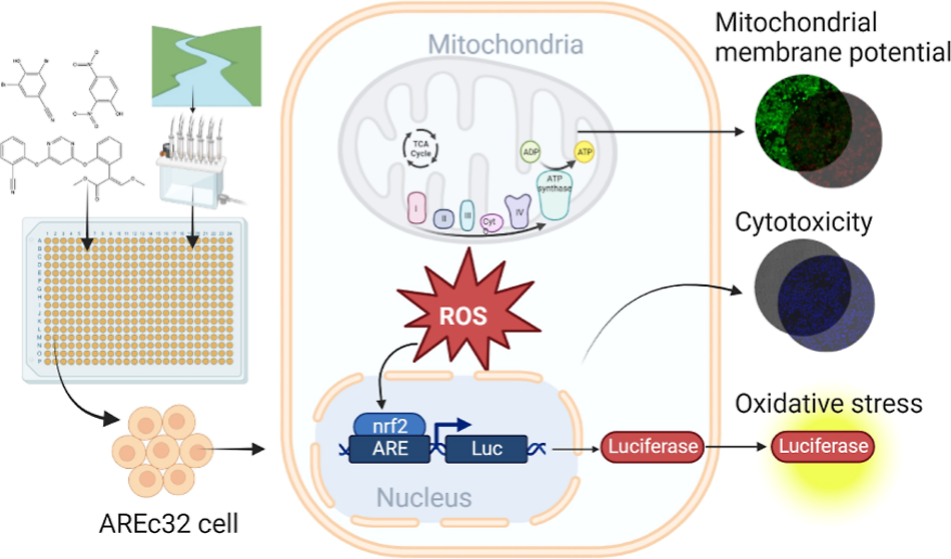

Mitochondria play a key role in the energy production of cells, but their function can be disturbed by environmental toxicants. We developed a cell-based mitochondrial toxicity assay for environmental chemicals and their mixtures extracted from water samples. The reporter gene cell line AREc32, which is frequently used to quantify the cytotoxicity and oxidative stress response of water samples, was multiplexed with an endpoint of mitochondrial toxicity. The disruption of the mitochondrial membrane potential (MMP) was quantified by high-content imaging and compared to measured cytotoxicity, predicted baseline toxicity, and activation of the oxidative stress response. Mitochondrial complex I inhibitors showed highly specific effects on the MMP, with minor effects on cell viability. Uncouplers showed a wide distribution of specificity on the MMP, often accompanied by specific cytotoxicity (enhanced over baseline toxicity). Mitochondrial toxicity and the oxidative stress response were not directly associated. The multiplexed assay was applied to water samples ranging from wastewater treatment plant (WWTP) influent and effluent and surface water to drinking and bottled water from various European countries. Specific effects on MMP were observed for the WWTP influent and effluent. This new MitoOxTox assay is an important complement for existing *in vitro* test batteries for water quality testing and has potential for applications in human biomonitoring.

## Abstract 2

Environmental pollutants targeting mitochondria were characterized with a high-throughput imaging-based multiplexed assay that quantifies mitochondrial dysfunction, oxidative stress response, and cytotoxicity, and the assay was applied for water quality monitoring.

## 1 Introduction

Mitochondria act as powerplants for cells.^1,2^ Mitochondria produce energy in the form of ATP via oxidative phosphorylation (OXPHOS) using a transmembrane potential of hydrogen ions generated by the mitochondrial electron transport chain (ETC). Environmental pollutants can disrupt mitochondrial function in various ways: inhibition of the ETC, uncoupling of OXPHOS, or inhibition of the synthesis of ATP.^3^ When mitochondrial function is impaired, excessive reactive oxygen species (ROS) are generated. Hence, the production of ROS was considered as indicator of mitochondrial damage for mitochondrial toxicants.^4^ A decrease in the mitochondrial membrane potential (MMP) has also been frequently used as a key indicator of mitochondrial dysfunction.^4−6^ Many recent epidemiological and experimental research works have provided evidence that exposure to environmental toxicants such as endocrine disruptors and pesticides could be linked to changes in biomarkers for mitochondrial damage (e.g., oxidative damage, MMP, and ATP levels).^7^ For example, bisphenol A induced oxidative stress and decreased MMP in lymphoblasts from children.^8^ Legradi et al. evaluated the potential of environmentally relevant hydroxylated polybrominated diphenyl ethers to disrupt OXPHOS using a rat mitochondria respiration assay and a MMP assay with fish cell lines.^9^

Due to the high environmental relevance of mitochondrial toxicity, it would be desirable to monitor the level of mitochondrial toxicants in environmental mixtures and extracts from biota and blood in biomonitoring studies. There have been attempts to monitor water quality based on oxygen consumption rate as an endpoint for mitochondrial toxicity using isolated mitochondria from bovine heart^10^ or HepG2 cells.^11^ To date, the quantification of the oxygen consumption rate remains limited to low-throughput systems in the 24-well or 96-well plate format.

The Tox21 program established an MMP assay multiplexed with cytotoxicity in HepG2 cells for high-throughput screening of Tox21 library compounds.^5,6^ It was reported that 11% of around 10,000 compounds decreased MMP without cytotoxic effects. Here, we adapted the high-content imaging and HTS MMP assay developed for testing individual chemicals in Tox21 and multiplexed it with a reporter gene assay that quantifies the oxidative stress response (AREc32) in a single 384-well plate for a more comprehensive evaluation of the mitochondrial toxicity of environmental pollutants and complex mixtures extracted from environmental samples.

Mitochondrial dysfunction-related ROS production may trigger the oxidative stress response, namely the Nrf2-mediated oxidative stress response pathway.^12^ The AREc32 assay quantifies the induction of the transcription factor Nrf2-mediated oxidative stress response pathway via the reporter protein luciferase.^13^ It has been reported that Nrf2 could be involved in the regulation of MMP,^14^ which means that the AREc32 assay can provide another endpoint for mitochondrial health. For example, the upregulation of the Nrf2-mediated pathway was assessed in HepG2 cells for in-depth investigation after primary screening using the MMP assay in the Tox21 mitochondrial toxicity study.^4^ The AREc32 assay has advantages in the context of environmental monitoring as it already has a long tradition of applications to water quality monitoring.^15,16^

The aim of this study was to implement a multiplexed assay of mitochondrial dysfunction, oxidative stress response, and cytotoxicity—called MitoOxTox—for water quality monitoring. Previous screening studies compared only cytotoxicity and MMP disruption to quantify the specificity of effects on MMP.^5,6^ However, further in-depth analysis is necessary to further distinguish cytotoxic effects from baseline cytotoxicity.^17^ Baseline toxicity is the minimal toxicity of a chemical caused by membrane intercalation^18,19^ and uncoupling transitions seamlessly into baseline toxicity for low-potency mitochondrial toxicants.^20^ The comparison between predicted baseline toxicity and experimental effects and cytotoxicity enables us to identify if specific MOAs trigger MMP disruption or even enhance cytotoxicity.^17,21^

The validity and robustness of the MitoOxTox assay were evaluated by testing single chemicals with diverse molecular initiating events (MIE) in mitochondria, among them numerous environmentally relevant fungicides. Then, the MitoOxTox assay was applied to surface water and wastewater treatment plant (WWTP) effluent samples that have been previously characterized with chemical analysis and large panels of bioassays.^22^ Mitochondrial toxicity was compared with the oxidative stress response and measured cytotoxicity as well as predicted baseline toxicity to evaluate the specificity of the mixture effect. Iceberg modeling was applied to connect the effects of the single chemicals to the measured effects of the extracts and to quantify the contribution of detected environmental pollutants.

## 2 Materials and Methods

### 2.1 Chemicals

A total of 33 mitochondrial toxicants with diverse MIEs were investigated in this study: 8 mitochondrial complex I inhibitors, 5 complex II inhibitors, 10 complex III inhibitors, 1 complex V inhibitor, 7 uncouplers, and 2 chemicals with multiple target sites in mitochondria. Additionally, three baseline toxicants (2-butoxyethanol, 4-chloro-3-methylphenol, and 4-pentylphenol) were tested for comparison. All tested chemicals are listed in Table S1 with their chemical identifiers, MIE, and physicochemical properties. Pentachlorophenol and azoxystrobin were used as positive controls for the MMP assay and *tert*-butylhydroquinone (tBHQ) for the oxidative stress response.

### 2.2 Surface Water and WWTP Effluent Extracts

In 2019, 85 surface water samples were collected during rain events in small streams close to German agricultural areas. From 2017 to 2019, 55 WWTP effluent samples were collected from 15 different European countries. These samples were extracted with solid-phase extraction (SPE) with Chromabond HR-X cartridges (Macherey-Nagel, Düren, Germany). Effect recovery for SPE extraction was good for water samples in 6 bioassays in previous studies with spiked samples.^23,24^ In addition, the chemical recovery of 251 organic compounds was investigated, and 159 chemicals had acceptable recoveries ranging from 60 to 123%. The chemical recoveries for the mitochondrial toxicants tested in the latter study were as follows: boscalid with 115.9%, azoxystrobin with 98.7%, trifloxystrobin with 20.1%, bromoxynil with 75.5%, and 24DNP with 67.1% (average: 75.5%).^24^ The solvent of SPE extracts was blown down, and the extracts were redissolved in MeOH with enrichment factors (*L*_water_/*L*_extract_) from 250 to 1000. The details of the samples and sampling method were already described by Liess et al.^25^ The details of the extraction/analytical method and the analytical results of individual samples can be found in Lee et al.^22^ for the surface water samples and in Finckh et al.^26^ for the WWTP effluents. Among the original set of samples, 20 surface water extracts and 20 WWTP effluent extracts were selected. The selection was based on the sum of the detected concentrations of fungicides, which are known to target mitochondria. 10 samples with the highest concentration of fungicides and another 10 samples with the lowest were considered; hence, a total of 20 water extracts were considered for each type of sample to cover a wide range of effects on MMP. The concentrations of the extracted environmental samples were expressed as the relative enrichment factor (REF, *L*_water_/*L*_bioassay_).

### 2.3 Cell Selection and Culture

Both ARE-bla and AREc32 cells are widely used for testing the oxidative stress response of chemicals. AREc32 cells were preferred over ARE-BLA in this study because the oxidative stress response was captured in AREc32 for more toxicants than for ARE-bla cells, and the response to the reference compound tBHQ was more robust and consistent over different plates in AREc32 (data not shown). The AREc32 cell line was provided by courtesy of C. Roland Wolf, Cancer Research, UK, and was maintained as described by Escher et al.^15^ The AREc32 cells were cultured in growth medium containing 90% of Dulbecco’s modified Eagle’s medium (Gibco, 31966-021) and 10% of fetal bovine serum (Gibco, 10099-141) with 1 mg/mL Geneticin (Gibco, 10131-035), 100 U/mL penicillin, and 100 μg/mL streptomycin (Gibco, 15140-122). The cells were incubated at 37 °C and 5% CO_2_ and were passaged every 2–4 days. The assay medium was prepared in the same way as the growth medium, except that it did not include geneticin. The cells were seeded at a density of 10,000 cells/well in a black wall/clear bottom (Corning, 3764) and incubated for 24 h. All experiments presented here were run with the black wall/clear bottom plates, but the process was successfully simplified to a white wall/clear bottom 384-well plate (Greiner, 781944), as discussed in the Supporting Information, Text S1.

### 2.4 Exposure to Chemicals/Samples

Methanol stocks were prepared for individual chemicals, and stocks were vortexed and sonicated until no precipitates were observed. The methanol stocks of chemicals and water extracts were either blown down under nitrogen or directly added into the assay medium up to a final concentration of 1% methanol to prepare the dosing medium. The aqueous solubility was retrieved from the EPA CompTox Chemicals Dashboard (https://comptox.epa.gov/dashboard), and enhancement of solubility by medium proteins^27^ was considered. The prepared dosing medium was further serially diluted into 11 test concentrations and added with two technical replicates to the 384-well plate plates containing cells using a pipetting robot (Hamilton Star, Bonaduz, Switzerland). The cells were exposed to the chemicals/samples for 24 h before the quantification of bioassay endpoints. The experiment was repeated in at least three independent experimental runs for chemicals/samples which were active in the first test run.

### 2.5 Image Acquisition and Analysis

The cell images were acquired by using two imaging devices according to a workflow outlined in Figure S1: ImageXpress High-Content Imaging System (Molecular Devices, Sunnyvale, CA, USA) and the IncuCyte S3 live cell imaging system (Essen BioScience, Ann Arbor, Michigan, USA). Previously, cell confluency has been measured as an estimate for cell viability.^18^ To verify comparability with the measurement using ImageXpress, cell confluency was measured additionally and analyzed by an IncuCyte S3 live cell imaging system for single compounds (Essen BioScience, Ann Arbor, Michigan, USA), as described by Escher et al.^18^ For the water samples, cytotoxicity from ImageXpress was recorded because it can be measured in one run with the MMP endpoint.

To acquire images using the ImageXpress, cells were loaded with Hoechst 33342 (Invitrogen, H3570) and the MMP indicator (*m*-MPI; Codex BioSolutions, CB-80600).^28^ The *m*-MPI dye was reported to have the highest signal-to-background ratio compared to other conventional dyes (JC-1, rhodamine 123, and tetramethylrhodamine), and the sensitivity was demonstrated with positive controls such as rotenone and antimycin.^29^ The detection mixture was prepared by the addition of Hoechst 33342 (final concentration in a well plate: 1 μg/mL) and *m*-MPI (1000× solution diluted into 1× in a well plate) into phosphate-buffered saline (PBS). Then, 20 μL of the detection mixture was added to each well of the assay plates using a multichannel pipet. After incubation for 30 min, phase-contrast and fluorescence images were acquired with a 10× objective lens in 4 different channels (transmitted light; 560 nm excitation/624 nm emission for red fluorescent aggregates of *m*-MPI; 475 nm excitation/536 nm emission for the green-fluorescent monomer form of *m*-MPI; 377 nm excitation/447 nm emission for Hoechst 33342). The images were further processed with a combination of the open-source software CellProfiler (ver 4.2.5)^30^ and the generalist, deep learning-based cellular segmentation method CellPose.^31^ First, cells were segmented based on phase-contrast images with the support of a nuclear channel (Hoechst 33342) using the Cytoplasm 2.0 model in CellPose. Then, the red and green fluorescence signals from the *m*-MPI dye were quantified within individual cells based on the cell mask. The red/green fluorescence ratio was calculated to estimate effects on MMP using eq 1.^28,29^
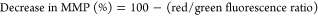
1

### 2.6 Luciferase Assay

After the acquisition of images on the assay plates, the cells were washed with PBS using a microtiter plate washer (BioTek, Winooski, Vermont, USA). Then, 10 μL of lysis buffer was added into each well with a multichannel pipet, and the plate was shaken at 1500 rpm for 20 min. Substrate buffer containing D-luciferin (AAT Bioquest, ABD-12506) was added to individual wells, and the plates were shaken again for 30 s. The cell lysate-substrate mix was transferred to a white wall/clear bottom 384-well plate (Corning, 3765) by using the pipetting robot. The luminescence was measured using a Tecan Infinite M1000 plate reader.

### 2.7 Data Evaluation

Concentration–response relationships for cytotoxicity and MMP disruption were considered for individual chemicals or samples to quantify concentrations causing a 10% effect. The inhibitory concentration for a 10% reduction in cell viability was determined as IC_10_, and the effect concentrations for a 10% reduction of the MMP was determined as EC_10_. In case of oxidative stress, the effect concentration leading to an induction ratio (IR) of 1.5 (EC_IR1.5_) was derived, as described by Escher et al.^15^

Automated data evaluation was performed using R software (version 4.1.3). Linear regression and log–logistic models were applied for each chemical/sample, and the respective R scripts and detailed explanations for the data processing are available on GitLab: https://git.ufz.de/braung/automatedbioassayscreening. A four parameter log–logistic concentration response model was used for the calculation of effect concentrations IC_10_ and EC_10_ corresponding to the absolute 10% effect using the tcpl R package.^32^ Concentrations above IC_10_ of cytotoxicity were excluded from the linear concentration–effect curves of the reporter gene activation for the derivation of EC_IR1.5_.^33^ This strict cutoff is necessary due to the problem of the cytotoxicity burst in reporter gene assays.^34^ In the case of mitochondrial toxicity, this cutoff was relaxed to 10 × IC_10_, i.e., when EC_10_ for MMP decrease was 10 times higher than IC_10_, the measured effects on MMP were considered secondary effects of cytotoxic effects, and no EC_10_ was derived.

### 2.8 Specificity Analysis

The specificity of effects was calculated using toxic ratios (TR) and specificity ratios (SR) as described previously.^17,21^ The toxic ratio TR compares measured cytotoxicity with predicted baseline toxicity (eq 2), which then serves as an indicator for an enhanced level of cytotoxicity.^34^ A higher TR would indicate that chemicals are more likely to have specific MOAs, other than baseline toxicity, that contribute to cytotoxicity. Chemicals with TR < 10 are typically classified as baseline toxicants, and those with TR > 10 are considered to have specific MOAs.^35,36^ Baseline toxicity is the minimal toxicity any chemical exhibits and refers to nonspecific effects on membranes due to the intercalation of the pollutants driven by their hydrophobicity.
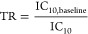
2

Baseline toxicity was predicted for individual chemicals with a prediction model from Lee et al.^37^ for the AREc32 cell line (eq 3). The distribution ratio between liposomes and water at pH 7.4 (*D*_lip/w_(pH 7.4)) was used for the prediction of the nominal concentration causing 10% cytotoxicity by baseline toxicity (IC_10,baseline_).
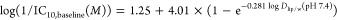
3

The specificity ratio SR indicates how specific the effects on MMP are compared to measured cytotoxicity (SR_cytotoxicity_; eq 4) and predicted baseline cytotoxicity (SR_baseline_; eq 5). SR_cytotoxicity_ can explain whether the observed decrease of the MMP was selective or just accompanied by cytotoxicity. Higher SR_cytotoxicity_ indicates that chemical MOAs act more specifically on a certain endpoint than on cell viability. SR_baseline_ gives a measure of how high the potency is compared to baseline toxicity. The higher SR_baseline_ of a chemical is, the more likely it is that the chemical has specific MOAs affecting endpoints of interest other than baseline toxicity. The SR approach has been already applied to other endpoints such as hormone receptor activation, oxidative stress response, and neurite outgrowth inhibition.^21,34^
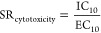
4
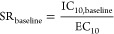
5

### 2.9 Iceberg Modeling

The measured effects of the samples in bioassays can be related to predicted effects using iceberg modeling.^17^ Bioanalytical equivalent concentrations (BEQ_bio_) are derived from bioassay measurements of samples and capture the entire mixture effect. Effect concentrations of each environmental sample were expressed as BEQ_bio_ compared to EC_10_ of 2,4-dinitrophenol (24DNP; eq 6).
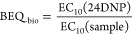
6

The predicted effects can be derived from the effect concentrations of the detected chemicals based on chemical analysis and the application of mixture models (BEQ_chem_). First, the EC ratio of 24DNP and chemical *i* can give relative effect potencies for each chemical *i* (REP_*i*_; eq 7). Second, the REP_*i*_ was multiplied by the detected concentration (*C*_*i*_) of chemical *i* to calculate BEQ_*i*_ for individual chemicals. Then, BEQ_chem_ can be calculated by summing up BEQ_*i*_ for all detected chemicals (eq 8). The BEQ concept assumes that chemicals with a common MOA behave in a concentration-additive manner in a mixture. This additive mixture model was successfully applied for many *in vitro* and *in vivo* assays^38−41^ and hence extended to MMP disruption in this study.
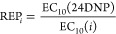
7
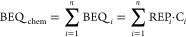
8

Both BEQ_chem_ and BEQ_bio_ were expressed in concentrations of 24DNP (i.e., 24DNP equivalent concentrations, 24DNP-EQ) so that they indicate concentrations of 24DNP that would induce the same effect as the mixture. By comparison of 24DNP-EQ_chem_ with 24DNP-EQ_bio_, the contribution of the individual detected chemical *i* to the overall mixture effect can be quantified (eq 9). The contribution of individual detected chemical *i* to the 24DNP-EQ_chem_ is defined by eq 10.
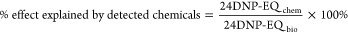
9
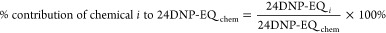
10

## 3 Results and Discussion

### 3.1 Measured Effects of Single Chemicals

The multiplexed MitoOxTox assay was validated with known mitochondrial toxicants. The AREc32 assay has been already applied to a large number of reference chemicals and environmental chemicals.^15,16^ Our test mainly focused on environmental chemicals that are known to target different sites in mitochondria, such as fungicides. In total, 33 mitochondrial toxicants with various underlying MIEs, and 3 baseline toxicants were tested.

Cytotoxicity IC_10_, EC_10_ for MMP inhibition, and EC_IR1.5_ for the activation of ARE were derived from concentration–response curves (CRC) of individual chemicals. An example of image analysis for cytotoxicity and MMP disruption is given in Figure S2. The CRCs of individual chemicals are shown in Figure S3, and the derived effect concentrations are given in Table S2. The IC_10_ for cytotoxicity measured with the newly optimized tool ImageXpress (Figure S1) did not deviate by more than a factor of 2.1 from those from the previously used imaging device IncuCyte (Figure S4 and Table S2). The positive control for oxidative stress response, tBHQ, showed stable CRCs (Figure S3) with robust EC_IR1.5_ when compared with those reported for the original AREc32 reporter gene assay (EC_IR1.5_ of 3.14 μM in the current study; 1.32 μM in Escher et al.^42^). Hence, the oxidative stress response in AREc32 provided the same assay quality even after multiplexing with the MMP measurement.

The strongest effects on cell viability and MMP were observed for the complex V inhibitor oligomycin A, with an IC_10_ of 1.8 nM and an EC_10_ of 0.24 nM. The lowest effects among the mitochondrial toxicants were observed for the complex I inhibitor carboxin, with an EC_10_ of 152 μM (no cytotoxicity). The baseline toxicant 2-butoxyethanol showed the lowest effects among all tested chemicals, with an IC_10_ of 13.6 mM and an EC_10_ of 10.7 mM. Among the Tox 21 compounds, the protein kinase C modulator bryostatin 1 decreased MMP with the highest potency, with an EC_50_ of 9.6 nM after 1 h of exposure in HepG2 cells.^6^

### 3.2 Cytotoxicity of Mitochondrial Toxicants

IC_10_ could be derived for 26 of a total of 33 mitochondrial toxicants and EC_10_ for 32 chemicals. In the case of complex II and III inhibitors, many chemicals were not impairing cell viability within the tested concentration range. Hence, for these two groups of chemicals, IC_10_ was only derived for 3 out of 5 complex II inhibitors and 6 out of 10 complex III inhibitors. The cytotoxicity for many of the tested mitochondrial toxicants was not specific with TR < 10 (Table S2). Cytotoxicity was specific with TR > 10 for only 2 out of 8 complex I inhibitors, none of complex II inhibitors, 1 out of 10 complex III inhibitors, 1 out of 2 multiple target sites, and 2 of 7 tested uncouplers. The complex V inhibitor, oligomycin A, had an exceedingly high TR of 19,461. The baseline toxicants fulfilled expectations with TR from 1.0 to 1.3.

### 3.3 Inhibition of the MMP

Among the 33 known mitochondrial toxicants, 31 chemicals were active with EC_10_ below IC_10_ (Table S2). SR_cytotoxicity_, which is a specificity measure for effects on the MMP compared to cytotoxicity, was available for the 26 mitochondrial toxicants: 7 out of 8 complex I inhibitors, 3 out of 5 complex II inhibitors, 6 out of 10 complex III inhibitors, 1 out of 1 complex V inhibitor, 2 out of 2 multiple target sites, and all 7 tested uncouplers.

The complex I inhibitors showed the highest effects on MMP among the tested mitochondrial toxicants (Figure S5). All 8 complex I inhibitors decreased MMP with high potency with EC_10_ below 13 nM (Table S2), while their cytotoxic effects expressed as IC_10_ were either similar or even weaker than those of other mitochondrial toxicants (Figure S5). This led to consistently pronounced SR_cytotoxicity_ for complex I inhibitors (mostly higher than 2000), while the toxicity of other mitochondrial toxicants was more variable even within the same receptor (Figure S5). SR_cytotoxicity_ of some mitochondrial toxicants (e.g., mepronil) was <10 despite their specific mechanism. Two uncouplers had SR_cytotoxicity_ < 10 but SR_baseline_ > 10 because the specific effect must have also directly affected the cell viability.

26 out of 33 mitochondrial toxicants in this study were also tested in the Tox21 MMP assay that applied to HepG2 cells.^4^ Both assays elicited a similar sensitivity. EC_50_ in HepG2 cells and EC_50_ in AREc32 cells were mostly within an order of magnitude (Figure S6), but Tox21 results were more than 10 times more sensitive with lower EC_50_ compared to MitoOxTox EC_50_ values for three chemicals: cyazofamid, dinoseb, and 24DNP. As cytochrome P450 (CYP) enzymes are mainly located in liver cells, these three chemicals could be metabolized into more potent MMP inhibitors in HepG2 cells, despite the fact that the HepG2 cells were only exposed for 1 h in the Tox21 MMP assay. Boscalid was consistently inactive for MMP inhibition in both assays, despite being known to be a complex II inhibitor. Fenazaquin was only active in the MitoOxTox assay, with an extremely high SR_cytotoxicity_ of 9356 (Table S2). Fenazaquin is a potent mitochondrial inhibitor that is detoxified by CYP2B6.^43^ As the HepG2 cells have the highest CYP2B6 activity,^44^ it is likely that fenazaquin was metabolized into less potent inhibitors for MMP. AREc32 is known to have inducible cytochrome P450 activity (CYP1A1)^45^ but not all chemicals can induce metabolic enzymes in AREc32. The high SR_cytotoxicity_ in the AREc32 cell line, which is based on MCF7 cells, indicates that AREc32 was not able to detoxify fenazaquin.

### 3.4 Relationship between Mitochondrial Toxicity and the Oxidative Stress Response

The oxidative stress response was only activated by 3 out of 33 mitochondrial toxicants: carboxin, 24DNP, and pentachlorophenol. The SR_cytotoxicity_ of pentachlorophenol was only 6.6, which means it was only marginally specifically activating the oxidative stress response, and carboxin was not cytotoxic. Interestingly, effects on MMP (EC_10_) and oxidative stress (EC_IR1.5_) started to appear at a similar concentration level for carboxin, pentachlorophenol, and 24DNP (Table S2), although the activation level of Nrf2 was not pronounced (Figure S5). In these cases, oxidative stress could be triggered as a result of MMP disruption.^14,46,47^ Considering 30 out of 33 mitochondrial toxicants showed no oxidative stress response, there seems to be no direct association between activation of the oxidative stress response and MMP disruption involved for these chemicals, despite their close relatedness in cellular toxicity pathways. Testing of chemicals with more diverse target sites could provide more evidence to connect those end points.

### 3.5 Relationship between Specific Effects and Cytotoxicity

**Figure 1:**
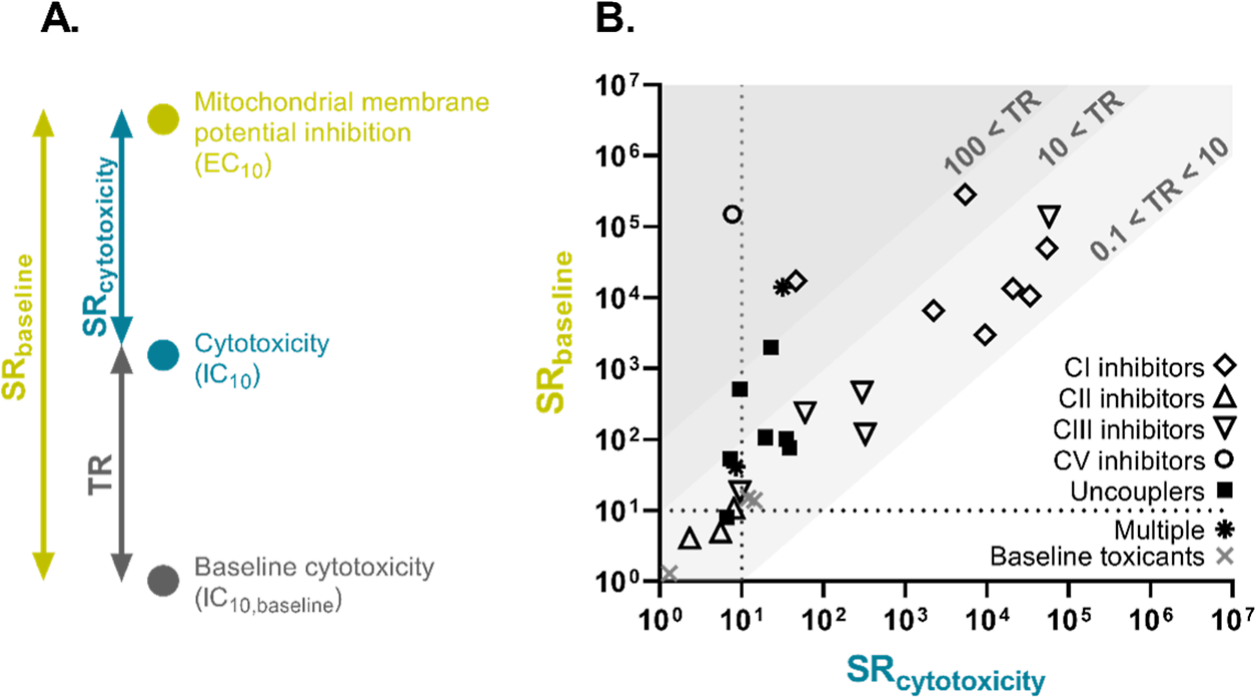
Specificity of effects on mitochondrial membrane potential (MMP) compared to cytotoxicity and baseline toxicity. (A) Visualization of the derivation of toxic ratio (TR; eq 2) and specificity ratios (SR; eqs 4 and 5) as the indicators of effect specificity. (B) SR_baseline_ plotted against SR_cytotoxicity_, and diagonally the thereof derived TR for all test chemicals (inhibitor of complexes I/II/III/V of the ETC, uncouplers, and baseline toxicants). The dashed line indicates specificity ratios of 10, which has been considered a threshold for effect specificity.^17,21^

The comparison of SR_cytotoxicity_ and SR_baseline_ in Figure 1 allows one to distinguish whether the MIE of these chemicals was highly specific without interfering with cytotoxicity or whether the effects were so severe that they directly reduced cell viability. The ratio of baseline and experimental cytotoxicity, i.e., TR, can measure how much more cytotoxic the chemicals were in comparison to baseline toxicity. The tested chemicals were grouped based on their MIEs in mitochondria: inhibition of complexes I/II/III/V of the ETC, uncoupling, and baseline toxicity.

The seven complex I inhibitors showed pronounced SR_cytotoxicity_ and SR_baseline_ compared with the other mitochondrial toxicants. The SR_cytotoxicity_ was all above 1000 except for rotenone (SR_cytotoxicity_ = 46). Despite their high SRs, the level of SR_cytotoxicity_ was similar to the corresponding SR_baseline_ for five of the complex I inhibitors, and this resulted in relatively low TR between 0.1 and 10. This would mean that the MMP disruption of complex I inhibitors was effective but did not directly lead to cytotoxicity. This was different for rotenone with a TR of 381 and berberine with a TR of 53.

The TR of the three complex II inhibitors ranged from 0.9 to 1.8, and the other two complex II inhibitors were not cytotoxic up to the highest tested concentrations. SR_baseline_ was derived for four complex II inhibitors and was relatively low, ranging from 4.1 to 10.8. This indicated that complex II inhibitors acted on MMP and cell viability just via their baseline toxicity, driven by their hydrophobicity.

Complex III inhibitors showed a wide distribution of SR_cytotoxicity_ and SR_baseline_ while also having low TR (Figure 1 and Table S2). Antimycin A showed extremely high SR_cytotoxicity_ of 57,489 and SR_baseline_ of 139,269, but another complex III inhibitor, hydramethylnon, only had SR_cytotoxicity_ of 0.3 and SR_baseline_ of 37.5. TR ranged from 0.4 to 4.0 for complex III inhibitors, except for hydramethylnon, which had exceptionally high TR of 145. Interestingly, the specificity ratios of complex III inhibitors tended to be higher for more hydrophilic ones with lower *D*_lip/w_ (Table S1 and S2). Hydrophobic chemicals are more likely to exert their toxicity via baseline toxicity due to their high affinity to membranes,^19^ which can support this observation. Also, the difference in chemical structure between the complex III inhibitors might also explain the wide range of specificity, considering the higher inhibition capacity of specific functional groups.^4^ This also corresponds to the previous study, which found that oxygen consumption rate was fully inhibited by antimycin A but not by other CIII inhibitors within the tested range.^48^

The complex V inhibitor oligomycin A had a remarkably high TR and SR_baseline_, while its SR_cytotoxicity_ was <10. This means that switching off complex V leads to a complete stop of ATP production, and the cell is not viable. Tributyltin acts as an uncoupler via the hydroxide/chloride antiport but also inhibits the ETC and ATP synthase.^49^ Tributyltin had the second highest toxic ratio (TR = 448) of all mitochondrial toxicants and also a relatively high SR_baseline_ (SR_baseline_ = 14,215), which could mean the inhibition of ATP synthesis and an even more severe effect on cell viability than complex V inhibition.

Uncouplers were also distributed over a wide range of SR_baseline_, but their SR_cytotoxicity_ was only up to 39, which was much lower than that of complex I inhibitors (Figure 1). This indicates that uncoupling depletes energy resources in the form of ATP so effectively that it directly leads to enhanced cytotoxicity. Of the chemicals that have multiple MIEs, bromoxynil mainly behaved as an uncoupler and not as an ETC inhibitor according to its SR_baseline_ and SR_cytotoxicity_.

Despite the fact that most of the mitochondrial toxicants showed either high TR (enhanced cytotoxicity) or high SR_cytotoxicity_ (specific effects on MMP), some mitochondrial toxicants, such as complex CII inhibitors, had both SRs as low as those from baseline toxicants. Similarly, a lower impact on the oxygen consumption rate, which is another measure for mitochondrial dysfunction, was observed for CII inhibitors in human renal and liver cells compared to CI and CIII inhibitors previously,^48^ possibly because its impact on the entire electron transfer chain could be attenuated by compensation with electron transfers from CI.

The results from single chemicals and comparison with the literature validate the MitoOxTox assay. This assay can capture a wide range of effect levels for MMP disruption and distinguish mitochondrial toxicants from baseline toxicants based on their specificity of MMP and excess cytotoxicity, but it also can differentiate between different MIEs of mitochondrial toxicity. If TR and SR_baseline_ are high, ATP synthesis seems to be affected through complex V inhibition, uncoupling, or ATP synthase inhibition. Inhibition of other components of the ETC appears to lead to high specificity of MMP but does not result in cytotoxicity (high SR_cytotoxicity_ and SR_baseline_ but low TR).

### 3.6 Application to Water Quality Assessment

To verify the robustness of the MitoOxTox assay for water quality assessment, surface water collected in Germany in small streams during rain events (*n* = 20) and diverse European wastewater treatment plant effluents (*n* = 20)^22^ were tested for their cytotoxicity, MMP disruption, and oxidative stress response in AREc32. Their IC_10_, EC_10_, and EC_IR1.5_ (Table S3) were derived from CRCs in Figure S7. The IC_10_ values from IncuCyte and from ImageXpress again agreed well (Figure S8A). Also, the IC_10_ and EC_IR1.5_ for oxidative stress responses aligned well between the conventional setup of the AREc32^22^ and our multiplexed assay (Figure S8B). The good agreement of the EC_IR1.5_ determined for the same set of samples from the previous study and this study (Table S3 in this study; Table S9 in Lee et al.^22^) confirmed again that the oxidative stress response assay can be multiplexed with MMP measurements (Figure S8B).

**Figure 2:**
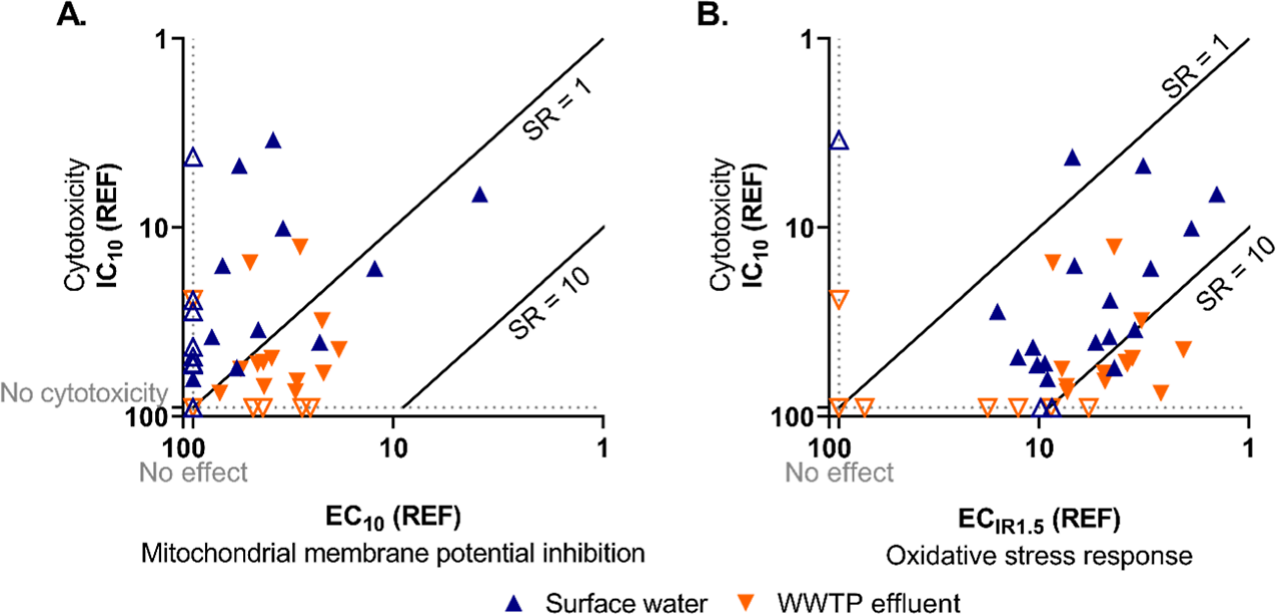
(A) Cytotoxicity versus mitochondrial membrane potential (MMP) disruption and (B) cytotoxicity versus oxidative stress response in AREc32 cells for surface water (blue upward triangles) and wastewater treatment plant (WWTP) effluent (orange downward triangles). The empty triangles are samples which did not show any effect up to the highest tested REF of 100.

WWTP effluent extracts showed more specific effects on MMP than surface water (Figure 2A). Only cytotoxicity or low specificity on MMP was observed for surface water (SR_cytotoxicity_ below or just above 1). Although EC_10_ could be derived from more WWTP effluent extracts, and many of them disrupted MMP more specifically with relatively higher SR_cytotoxicity_, the degree of specificity was still not pronounced also for WWTP effluent extracts (SR_cytotoxicity_ up to 2.7). Considering that the EC_10_ was determined to be close to the IC_10_ level for both types of water extracts, the observed MMP effects could also be a secondary effect of cytotoxicity, and only low concentrations of specific MMP disruptors might be present in the real water sample.

In the case of the oxidative stress response, higher specificity of effects was observed again for individual WWTP effluents with SR_cytotoxicity_ up to 28.7 (Figure 2B). This indicates that there might be more potent or higher concentrations of oxidative stress inducers in the WWTP effluent extracts than in surface water. According to Lee et al.,^22^ an industrial chemical, 2-benzothiazolesulfonic acid, was often detected in the WWTP effluent samples and was characterized as a main toxicity driver for the oxidative stress response.

### 3.7 Iceberg Modeling

Twelve of the mitochondrial toxicants that were characterized with the MitoOxTox were also on the target list for the chemical analysis of these samples.^22^ The complex II inhibitor boscalid was detected but had no effect on MMP up to 8.32 × 10^–5^ M. The complex I inhibitor pyridaben was an extremely potent inhibitor of MMP but was not detected in any of the water extracts. The uncouplers 24DNP, dinoseb, and bromoxynil were detected mainly in the surface water samples, and 24DNP was also detected in 4 WWTP effluent samples. 24DNP had often very high concentrations and high relative effect potency and was therefore chosen as a reference chemical to express the BEQ as 24DNP-EQ. Of the fungicides, azoxystrobin was detected most frequently in both water types, but the five other strobilurins (dimoxystrobin, fluoxastrobin, picoxystrobin, pyraclostrobin, and trifloxystrobin) also occurred occasionally.

**Figure 3:**
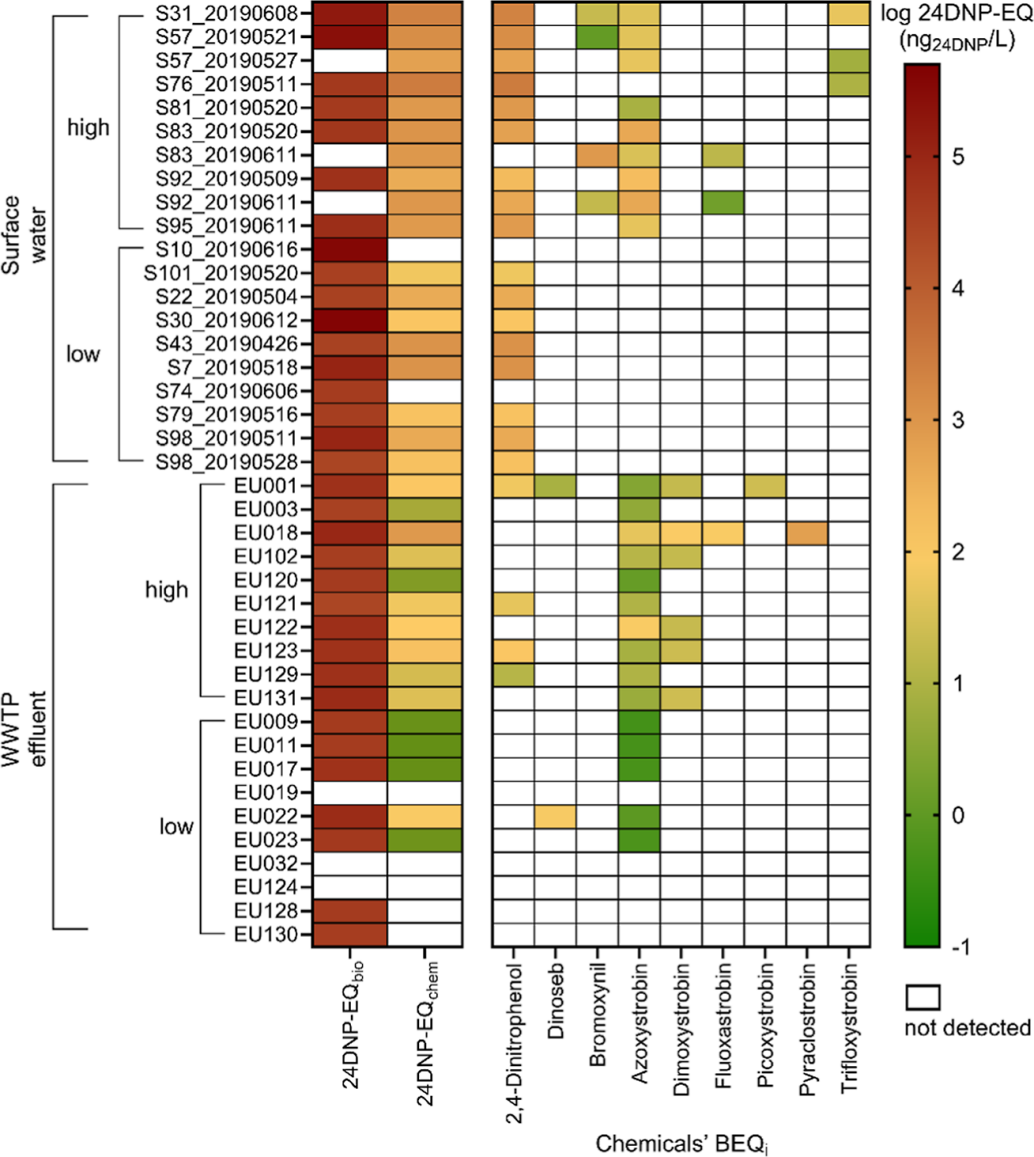
Contribution of the individual mitochondrial toxicants to the predicted mixture effect 24DNP-EQ_chem_ and comparison with the measured mixture effect 24DNP-EQ_bio_. EQ = equivalent concentration.

The EC_10_ of the MMP endpoint was converted to 24DNP-EQ_bio_. The 24DNP-EQ_bio_ values indicate the effects of the entire complex mixture and were high for several surface water samples, as expected from their low EC_10_ (Figure 3). When we compared 24DNP-EQ_bio_ with 24DNP-EQ_chem_, single chemicals that had measured toxicity information and were also detected in the samples explained up to 5.8% of the measured mixture effects in surface water (Figure S9A). The detected bioactive chemicals explained only up to 0.84% in the WWTP effluents, and most of the samples explained even less than 0.1%, which means that more toxicity information on single chemicals is required to better explain the effects observed in WWTP effluent. It appears that the % effect explained by the detected chemicals increased with the number of detected and bioactive chemicals (Figure S9B).

The mixture effects of 24DNP-EQ_chem_ on surface water were strongly influenced by 24DNP (Figure 3). For surface water samples in which 24DNP was detected, 24DNP dominated 24DNP-EQ_chem_, and the rest of the detected chemicals explained only small fractions (Figure 3 and Table S4). In the case of the WWTP effluent, azoxystrobin mainly contributed to 24DNP-EQ_chem_ but explained only up to 0.12% of the entire mixture effects expressed as 24DNP-EQ_bio_. In 5 WWTP effluent samples, dimoxystrobin explained a higher percentage of 24DNP-EQ_chem_ (up to 82% of 24DNP-EQ_chem_) compared to azoxystrobin, but 24DNP-EQ_chem_ covered merely up to 0.087% of 24DNP-EQ_bio._ In EU018, 24DNP-EQ_chem_ explained 0.84% of 24DNP-EQ_bio_, which was the highest among WWTP effluents. For EU018, pyraclostrobin covered 74% of 24DNP-EQ_chem_ (0.62% of 24DNP-EQ_bio_); hence, the contribution of diverse strobilurins to mitochondrial toxicity was observed in the WWTP effluent.

### 3.8 Outlook

We identified complex I inhibitors as highly specific toxicants acting on MMP. The MMP effects of more chemicals should be quantified to explain the observed effects of mixtures extracted from water samples. Many drugs have been reported to inhibit and uncouple the electron transport chain as a main off-target effect.^50,51^ Also, the Tox21 chemical screening listed the 20 most potent active compounds from the MMP screen using HepG2 cells, which ranged from several fungicides, dyes, and pharmaceuticals to a few industrial chemicals.^6^ Hence, diverse chemicals can play a role in mitochondrial toxicity in the environment. Chemicals that are frequently detected in the environment would have high priority for testing to facilitate the identification of toxicity drivers through iceberg modeling.^16,52^ The developed assay can not only be used for testing the mixture toxicity of water samples but also can be applied to other complex mixtures from biota and human samples. The toxicity information derived in this study can also be applied to identify toxicity drives in other types of mixtures.

Cells can have a lower sensitivity to mitochondrial toxicants in a high-glucose medium as their ATP generation can also rely on glycolysis. Especially cancer cells could be metabolically reprogrammed, which can lead to enhanced aerobic glycolysis.^53^ Low glucose culture conditions, which are often used for testing mitochondrial toxicity to increase sensitivity, were reported to induce reactive oxygen species in many cells.^54,55^ To avoid false positive effects from the oxidative stress response in our MitoOxTox assay, a normal assay medium with high glucose was used. Considering that the response for MMP disruption in the MitoOxTox assay was still similarly sensitive for the tested mitochondrial toxicants compared to Tox21 data, the MitoOxTox assay has enough capacity for capturing MMP disruption effects despite its high glucose condition. However, it should be further investigated how the responses of two endpoints in the MitoOxTox assay, i.e., the oxidative stress response and MMP disruption, differ between high and low glucose condition.

On one hand, evaluation of MMP can capture a wide range of mitochondrial toxicants by covering chemicals with diverse MIEs since multiple MIEs could be involved in MMP disruption. On the other hand, specificity analysis such as SR_cytotoxicity_ herein is required for this key event-based assay since MMP disruption could be merely a secondary effect of cytotoxicity if it occurs at concentrations that are already cytotoxic. We can expect that responses directly related to MIEs are rather consistent across different cell lines, while key events are more likely to be dependent on cell types for MMP inhibition.^56^ As mentioned above, cancer cells, including AREc32 cells herein, have reduced OXPHOS capacity and enhanced glycolysis,^57,58^ which means that primary/stem cells could potentially improve the sensitivity of the MMP assay. Hence, the selection of cell types and endpoints should be carefully chosen considering the purpose of mitochondrial toxicity assessment.

Mitochondrial dynamics have been investigated with an image-based approach for various morphological endpoints.^59,60^ For example, fragmentation of mitochondria was connected to MIEs of toxicants, e.g., inhibition of OXPHOS complexes, where effects on MMP and cytotoxicity were measured in parallel in the same image-based test system.^61^ The morphological changes can be even connected to chemical structure or other biological endpoints, such as gene expression.^62^ Hence, the current image-based approach can be easily extended with additional endpoints in future studies.

## Supporting Information Available

Additional information on chemicals, experimental details, CRC, and additional analyses (PDF)
Chemical identifiers, physicochemical properties, and mode of action of tested chemicals; constants; effect concentrations of single chemicals and water extracts for cytotoxicity, MMP disruption, and oxidative stress response in AREc32; and iceberg modeling for MMP disruption (XLSX)
