# Supplementary material for: Water Quality Monitoring with the Multiplexed Assay MitoOxTox for Mitochondrial Toxicity, Oxidative Stress Response, and Cytotoxicity in AREc32 Cells
Source: Environ Sci Technol. 2024 Mar 19;58(13):5716–26. doi: 10.1021/acs.est.3c09844 (PMC10993414; doi:10.1021/acs.est.3c09844)
Supplement: Supplementary file 1 — es3c09844_si_001.pdf [file es3c09844_si_001.pdf]

Supporting Information

Water quality monitoring with the multiplexed assay MitoOxTox for mitochondrial toxicity, oxidative stress response and cytotoxicity in AREc32 cells

Jungeun Lee†, Maria König†, Georg Braun†, and Beate I. Escher†‡ *

† Department of Cell Toxicology, UFZ–Helmholtz Centre for Environmental Research, 04318 Leipzig, Germany
‡ Environmental Toxicology, Department of Geosciences, Eberhard Karls University, Schnarrenbergstr. 94-96, 72076 Tübingen, Germany

4 tables, 10 figures, 1 additional text

Table of Contents

Table S1. Chemical identifiers, physicochemical properties, and mode of action of tested chemicals. 3

Figure S1. Experimental procedure for measurement of cytotoxicity, mitochondrial membrane potential (MMP) disruption and oxidative stress response using AREc32 cells. 3

Figure S2. Phase-contrast and fluorescence images from ImageXpress and cytoplasm masks generated by CellPose for non-exposed cells and cells after exposure to pentachlorophenol for 24 h. 4

Figure S3. Concentration-response curves of single chemicals for cytotoxicity, MMP disruption and oxidative stress response in AREc32 cells. 5

Table S2. Effect concentrations of single chemicals for cytotoxicity, MMP disruption, and oxidative stress response in AREc32 cells 12

Figure S4. Comparison of measured cytotoxicity from ImageXpress and IncuCyte for tested chemicals in AREc32 cells. 12

Figure S5. Effects of mitochondrial toxicants and baseline toxicants on cell viability and mitochondrial membrane potential (MMP) in AREc32 cells. 12

Figure S6. Comparison of EC_{50} values for MMP disruption derived from MMP assay using HepG2 cells in Tox21 and those from MitoOxTox assay using AREc32 cells in this study for commonly tested mitochondrial toxicants. 13

Figure S7. Concentration-response curves of surface water and wastewater treatment plant (WWTP) effluent for cytotoxicity, MMP disruption and oxidative stress response in AREc32 cells. 14

Table S3. Effect concentrations of surface water and wastewater treatment plant (WWTP) effluent for cytotoxicity, MMP disruption and oxidative stress response in AREc32 cells 22

Figure S8. (A) Comparison of cytotoxicity measured with ImageXpress and IncuCyte for water extracts in AREc32 cells. (B) Comparison of IC_{10} for cytotoxicity and $EC_{IR1.5}$ for the activation of oxidative stress response in AREc32 cells using the conventional setup and the new multiplexed MitoOxTox assay. 22

Figure S9. (A) Comparison of 24DNP-EQ_{bio} and 24DNP-EQ_{chem} for the MMP endpoint for surface water samples (blue circles) and WWTP effluents (red diamonds). (B) Relationship between number (#) of chemicals detected and 24DNP-EQ_{chem}. 22

Table S4. Iceberg modelling for MMP disruption of surface water and waste water treatment plant (WWTP) effluent. 22

Text S1. Simplification of experimental procedure. 23

Figure S10. Comparison of (A) IC_{10}, (B) EC_{10}, and (C) $EC_{IR1.5}$ between experiments using Greiner plates with white wall/clear bottom for all three endpoints and different plates for detection of the different endpoints. 24

Table S1. Chemical identifiers, physicochemical properties, and mode of action of tested chemicals – see Supporting Information excel file.

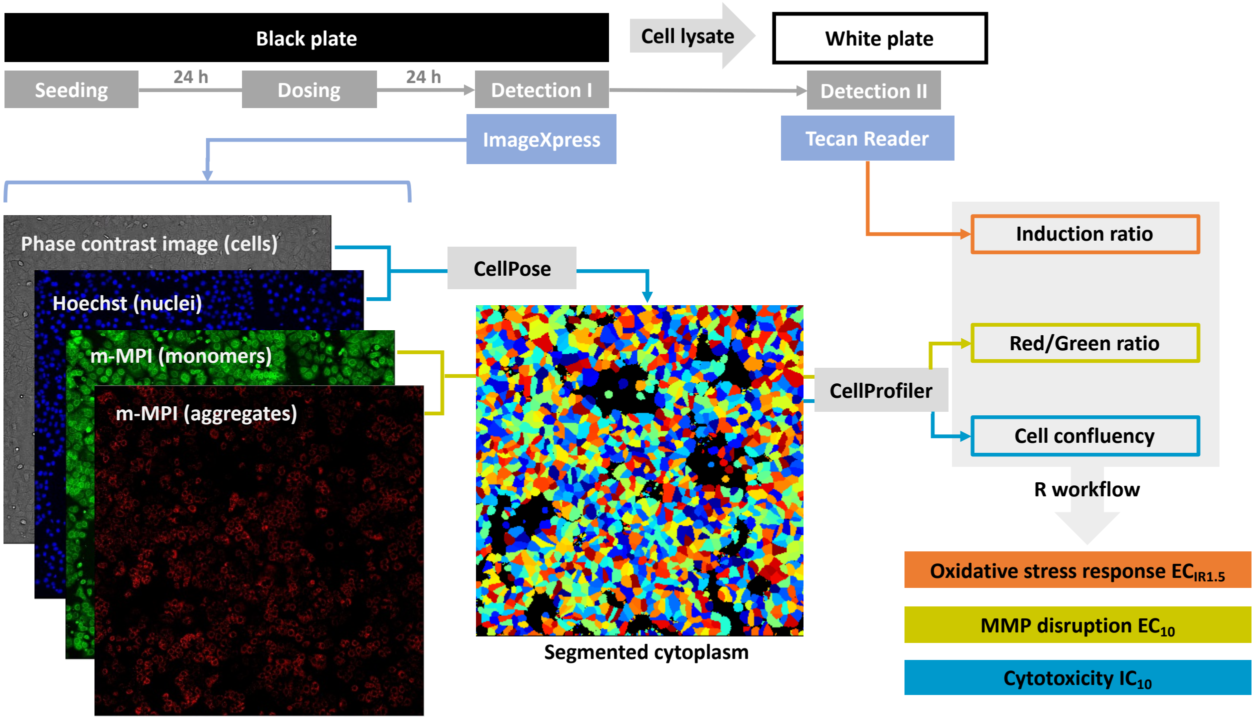

Figure S1. Experimental procedure for measurement of cytotoxicity, mitochondrial membrane potential (MMP) disruption and oxidative stress response using AREc32 cells.

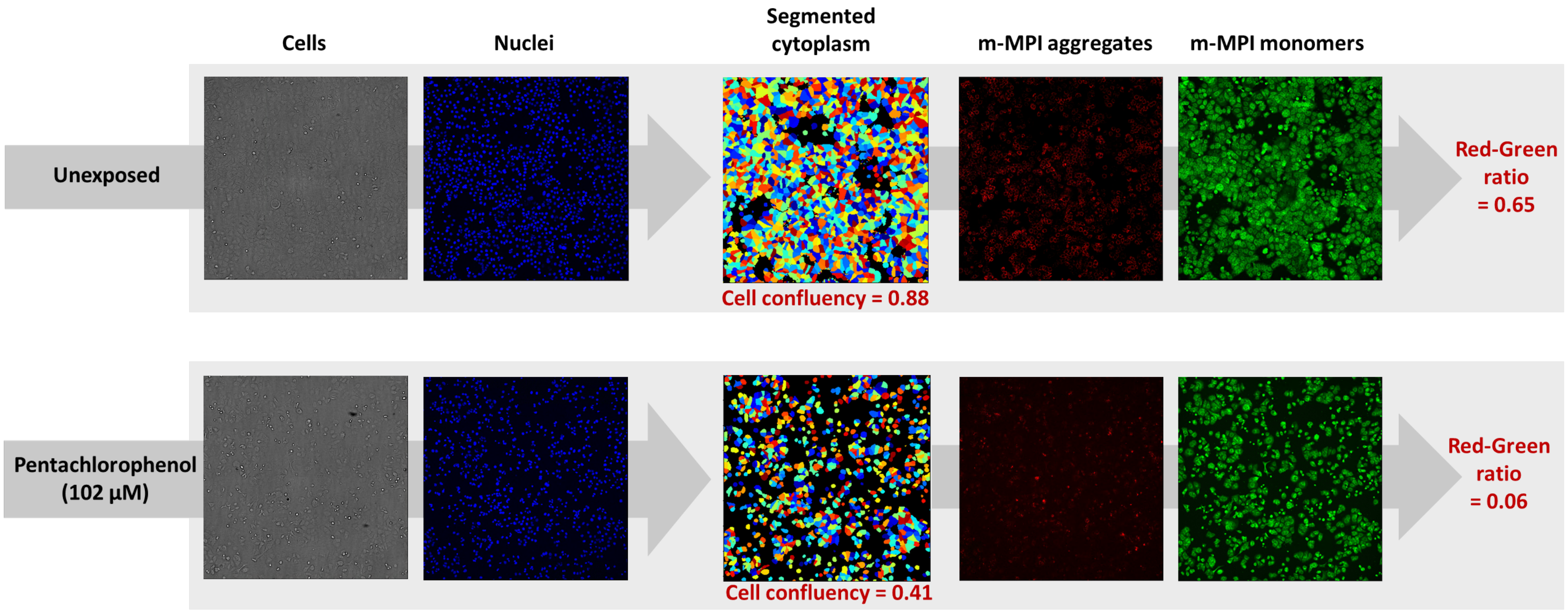

Figure S2. Phase-contrast and fluorescence images from ImageXpress and cytoplasm masks generated by CellPose for non-exposed cells and cells after exposure to pentachlorophenol for 24 h.

(a) Berberine

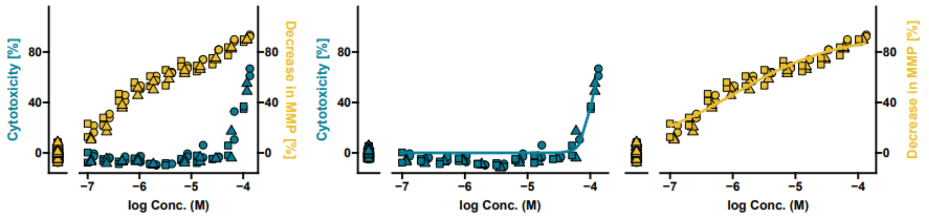

(b) Deguelin

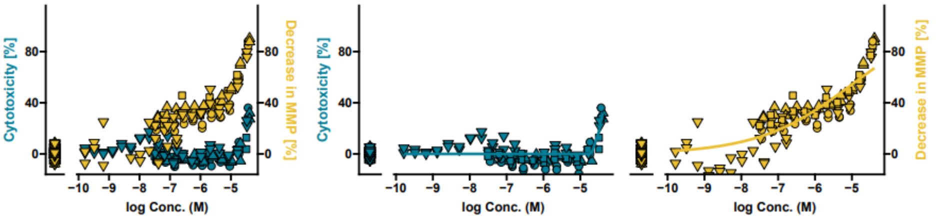

(c) Fenazaquin

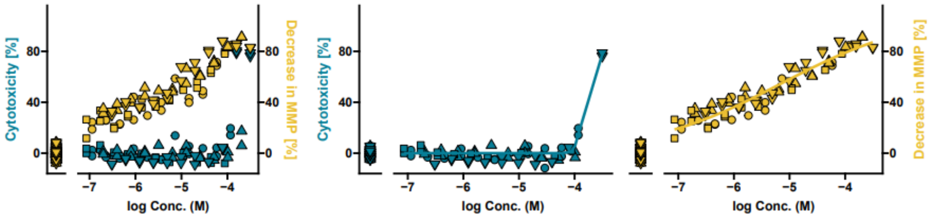

(d) Fenpyroximate

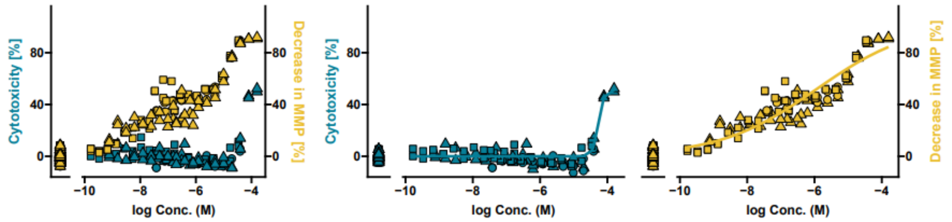

(e) Pyridaben

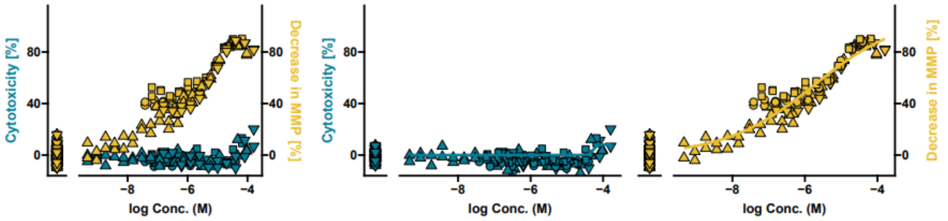

Figure S3. Concentration-response curves of single chemicals for cytotoxicity, MMP disruption and oxidative stress response in AREc32 cells.

(f) Pyrimidifen

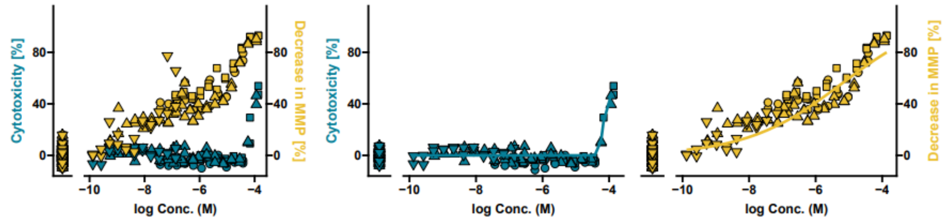

(g) Rotenone

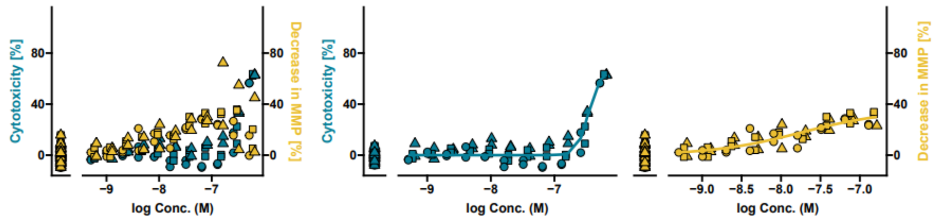

(h) Tebufenpyrad

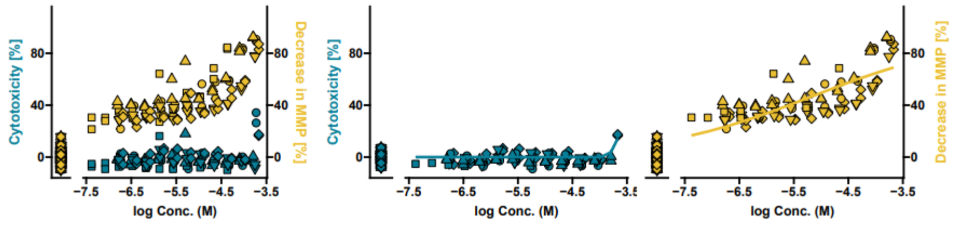

(i) Carboxin

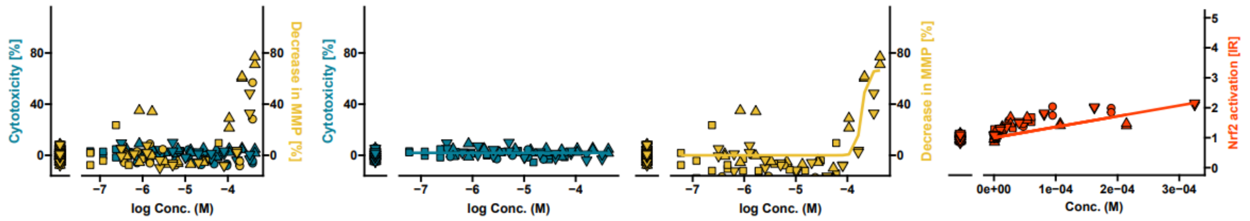

(j) Flutolanil

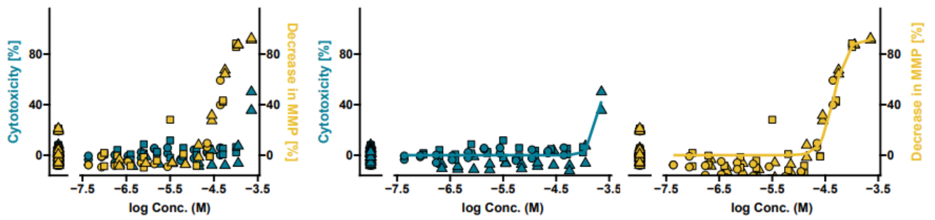

Figure S3. Continued. Concentration-response curves of single chemicals for cytotoxicity, MMP disruption and oxidative stress response in AREc32 cells.

(k) Mepronil

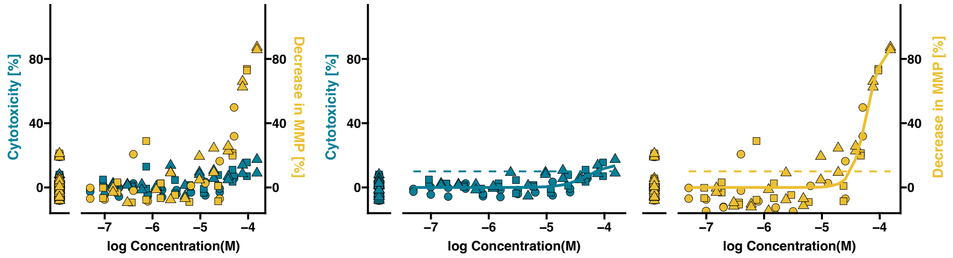

(l) Thifluzamide

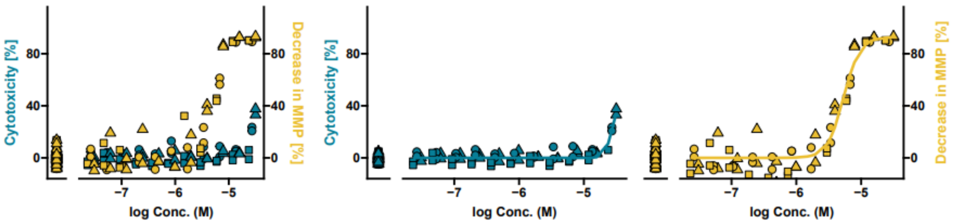

(m) Antimycin A

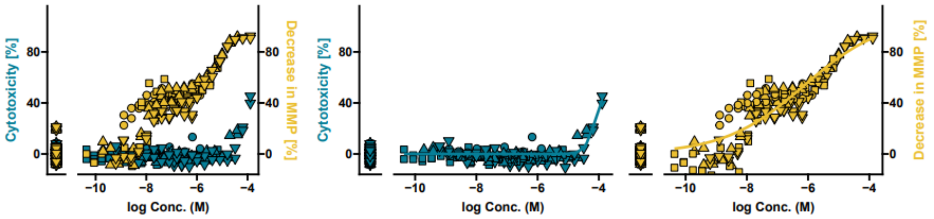

(n) Azoxystrobin

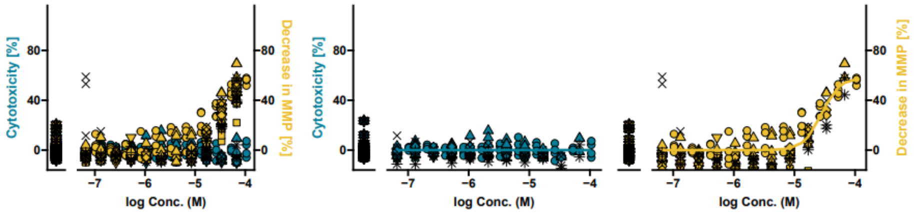

(o) Cyazofamid

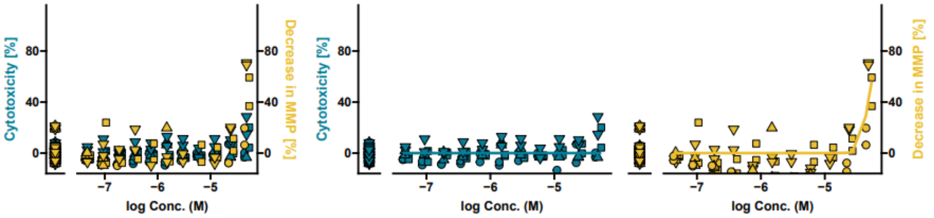

Figure S3. Continued. Concentration-response curves of single chemicals for cytotoxicity, MMP disruption and oxidative stress response in AREc32 cells.

(p) Dimoxystrobin

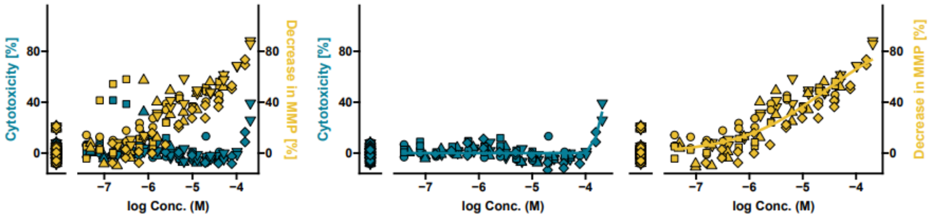

(q) Fenamidone

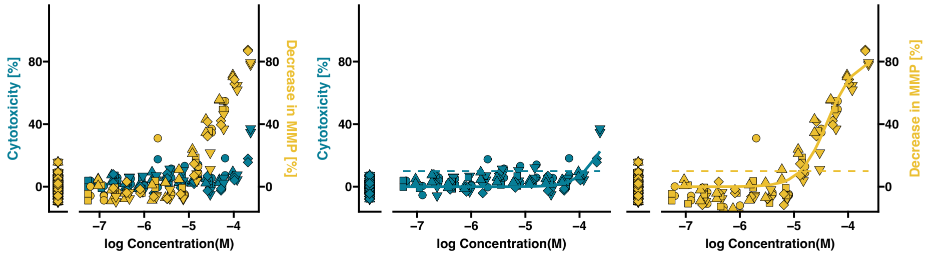

(r) Fluoxastrobin

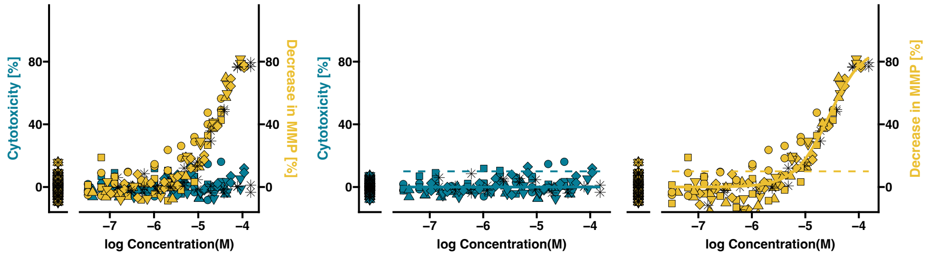

(s) Hydramethylnon

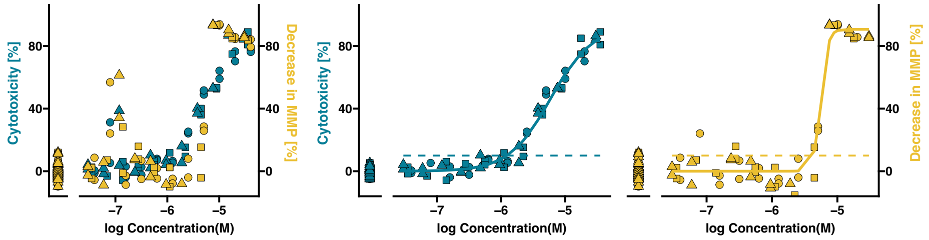

(t) Picoxystrobin

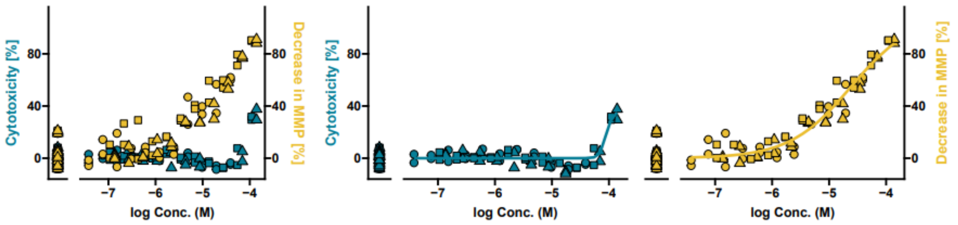

Figure S3. Continued. Concentration-response curves of single chemicals for cytotoxicity, MMP disruption and oxidative stress response in AREc32 cells.

(u) Pyraclostrobin

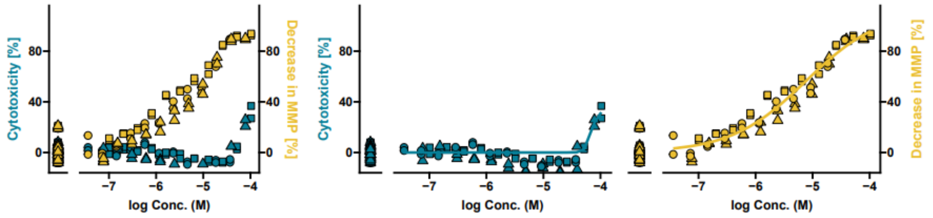

(v) Trifloxystrobin

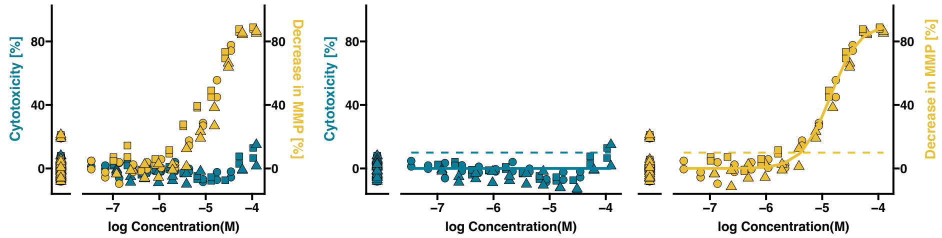

(w) Oligomycin A

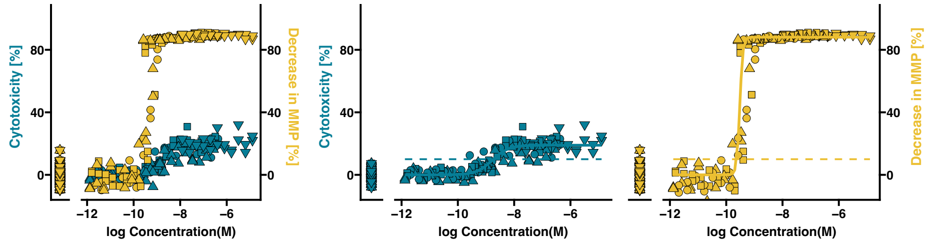

(x) Bromoxynil

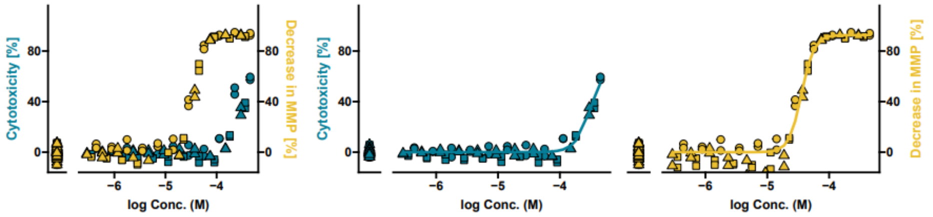

(y) Tributyltin Chloride

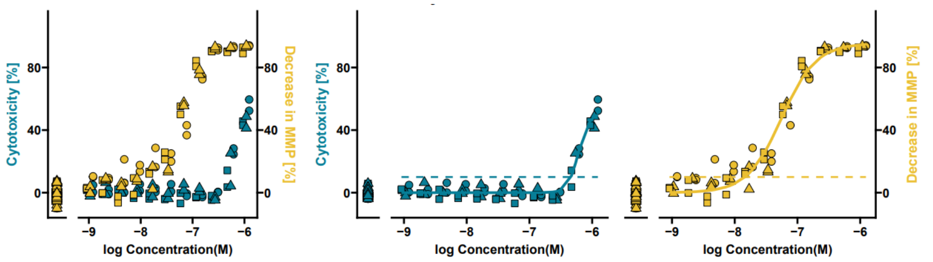

Figure S3. Continued. Concentration-response curves of single chemicals for cytotoxicity, MMP disruption and oxidative stress response in AREc32 cells.

(z) 2,4-Dinitrophenol

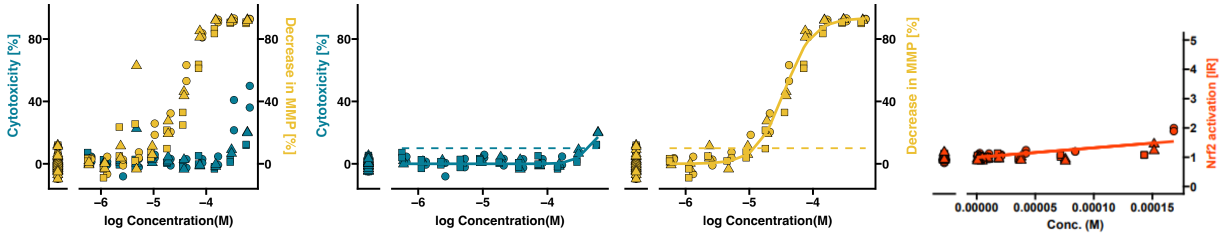

(aa) 4,6-Dinitro-o-cresol

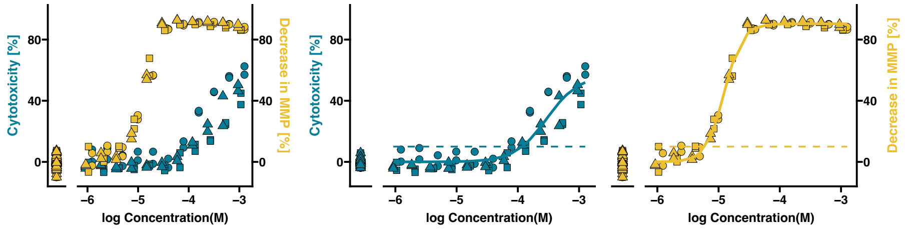

(bb) Carbonyl cyanide 3-chlorophenyl-hydrazone (CCCP)

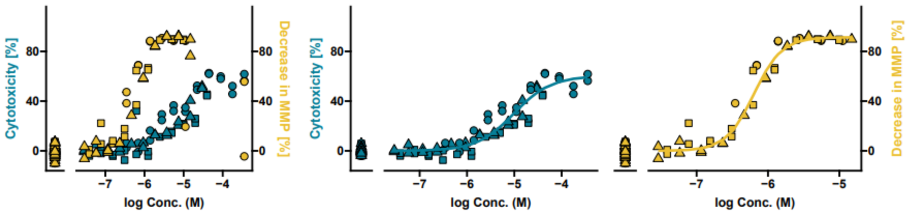

(cc) Carbonyl cyanide 4-(trifluoromethoxy)phenylhydrazone (FCCP)

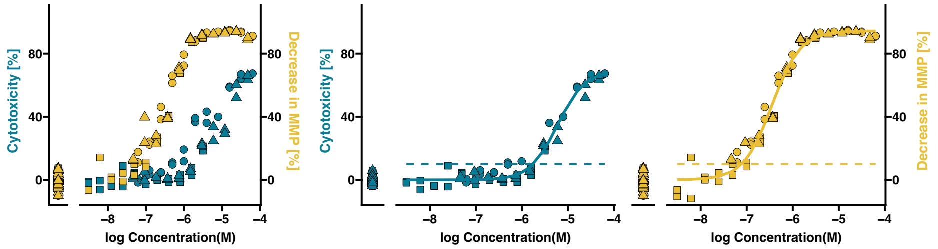

(dd) Dinoseb

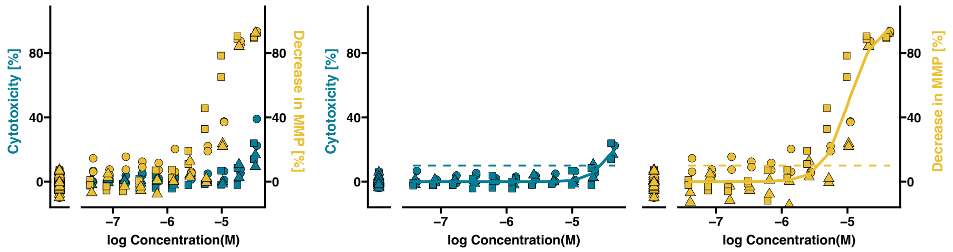

Figure S3. Continued. Concentration-response curves of single chemicals for cytotoxicity, MMP disruption and oxidative stress response in AREc32 cells.

(ee) Hexachlorophene

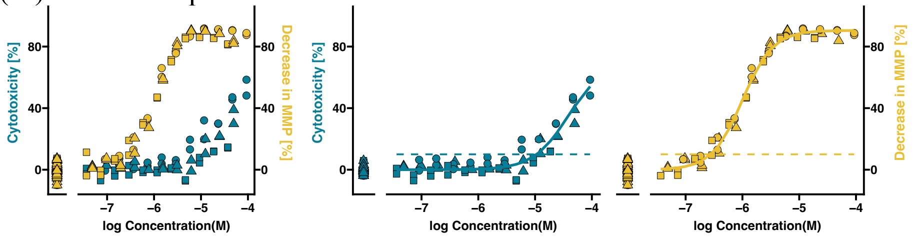

(ff) Pentachlorophenol

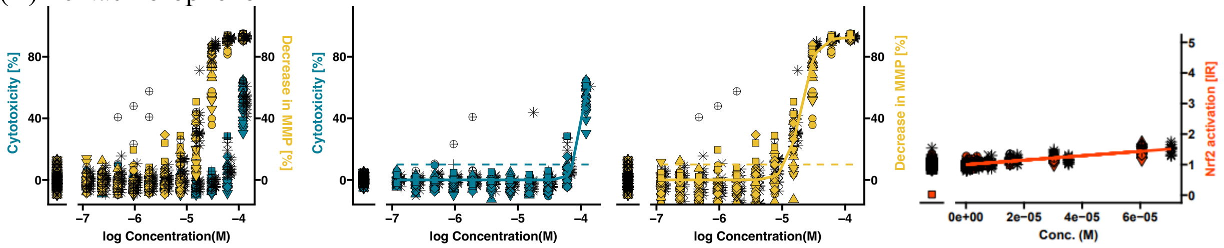

(gg) 2-Butoxyethanol

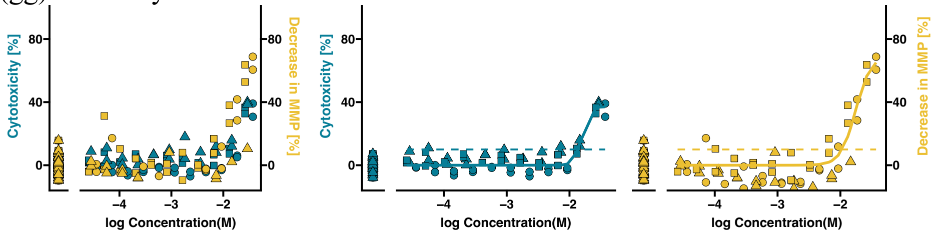

(hh) 4-Chloro-3-methylphenol

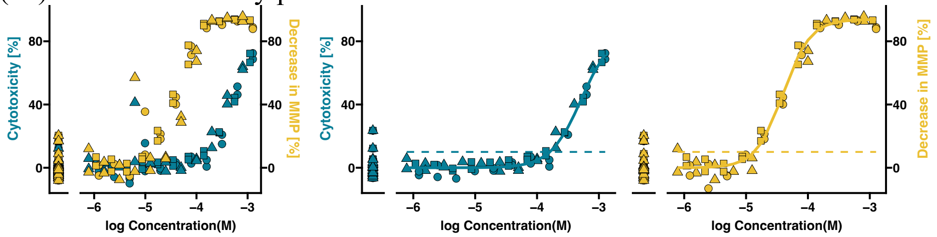

(ii) 4-Pentylphenol

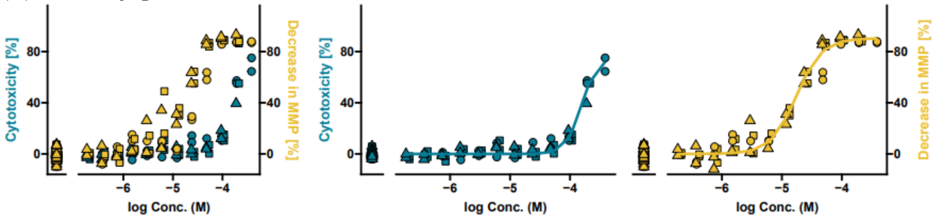

(jj) tBHQ

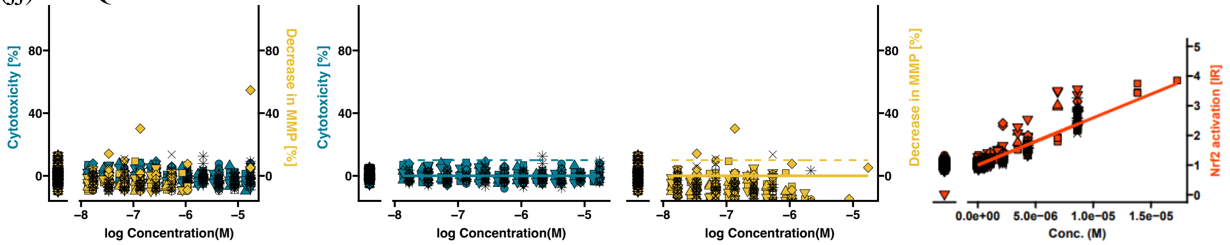

Figure S3. Continued. Concentration-response curves of single chemicals for cytotoxicity, MMP disruption and oxidative stress response in AREc32 cells.

Table S2. Effect concentrations of single chemicals for cytotoxicity, MMP disruption, and oxidative stress response in AREc32 cells – see Supporting Information excel file.

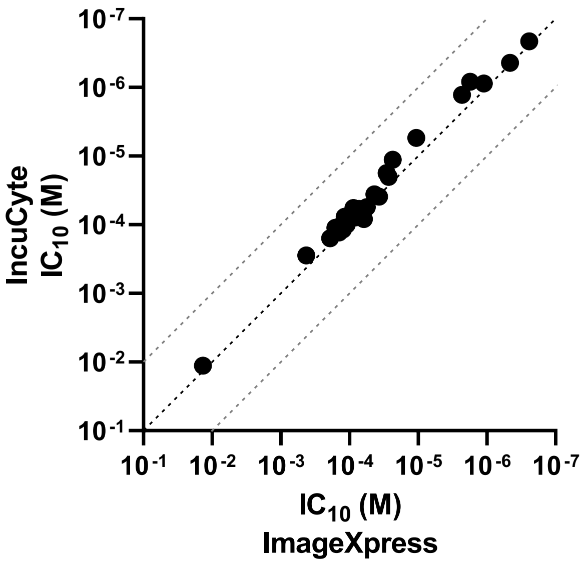

Figure S4. Comparison of measured cytotoxicity from ImageXpress and IncuCyte for tested chemicals in AREc32 cells.

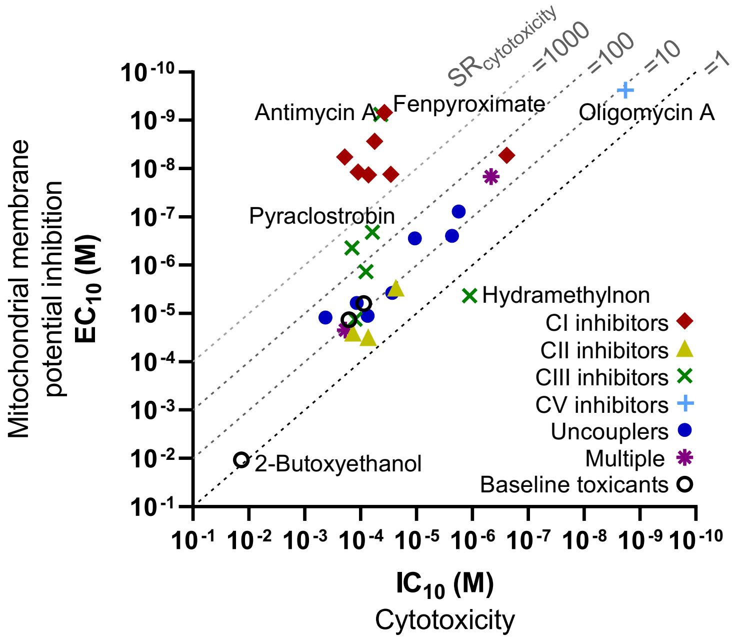

Figure S5. Effects of mitochondrial toxicants and baseline toxicants on cell viability and mitochondrial membrane potential (MMP) in AREc32 cells.

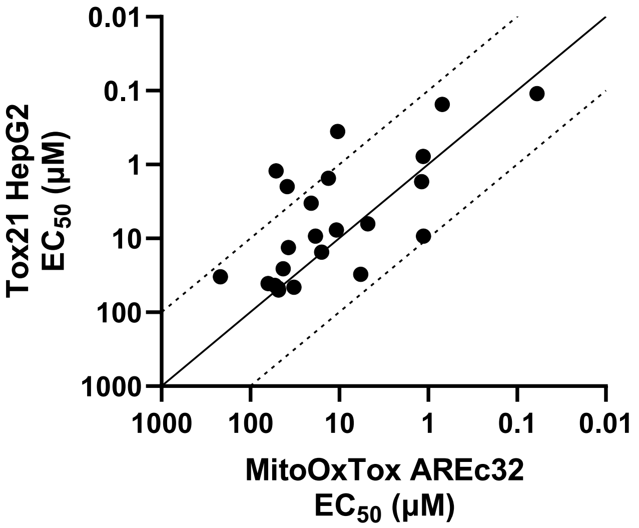

Figure S6. Comparison of EC_{50} values for MMP disruption derived from MMP assay using HepG2 cells in Tox21 (exposure duration for 1 h) and those from the 24h-MitoOxTox assay using AREc32 cells in this study for commonly tested mitochondrial toxicants. EC_{50} values for MitoOxTox assay were analyzed using log-logistic models.

Surface water

(a) S7_20190518

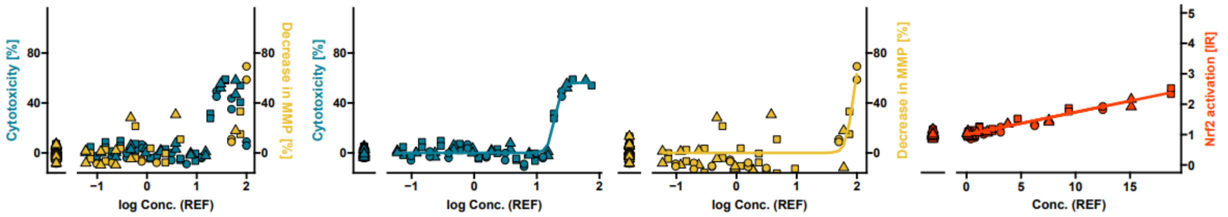

(b) S10_20190616

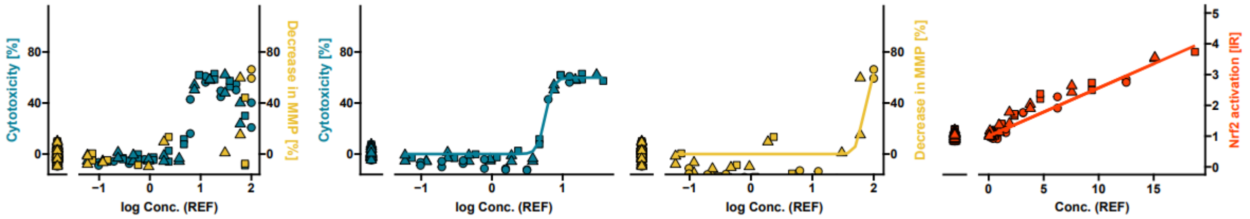

(c) S22_20190504

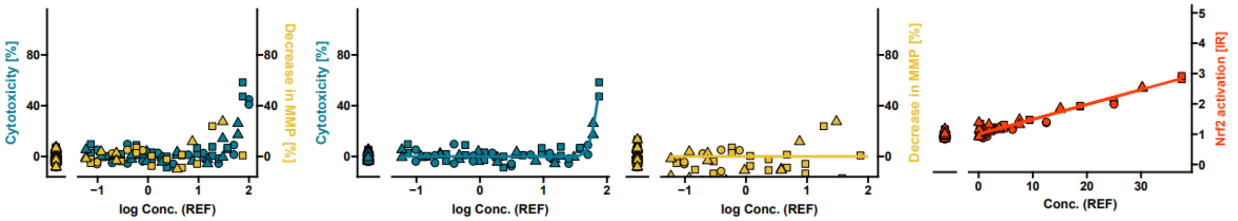

(d) S30_20190612

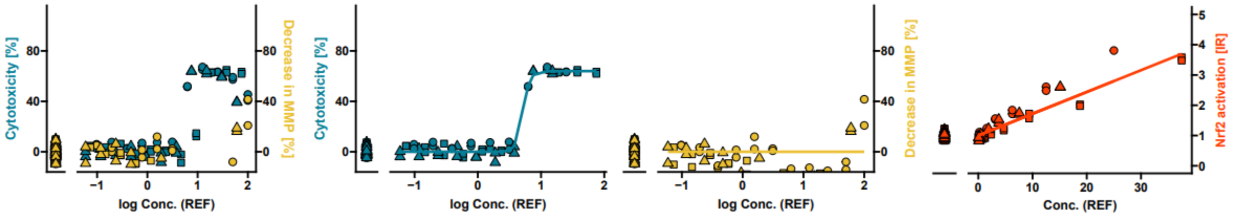

(e) S31_20190608

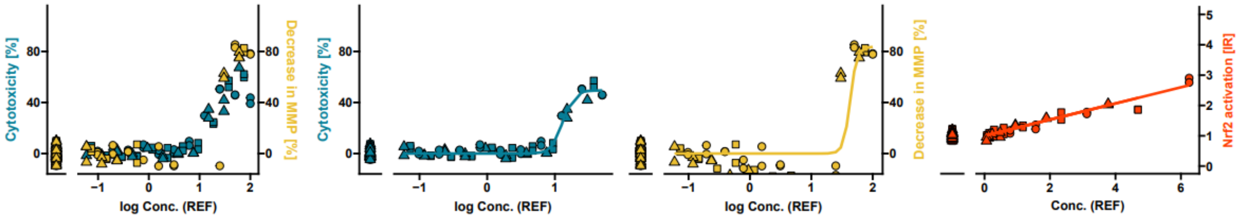

Figure S7. Concentration-response curves of surface water and wastewater treatment plant (WWTP) effluent for cytotoxicity, MMP disruption and oxidative stress response in AREc32 cells.

(f) S43_20190426

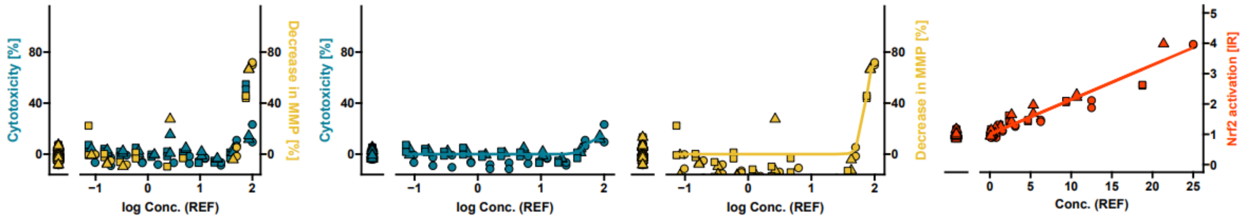

(g) S57_20190521

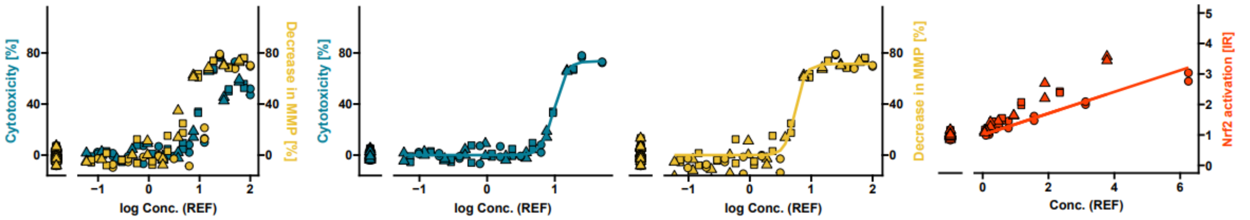

(h) S57_20190527

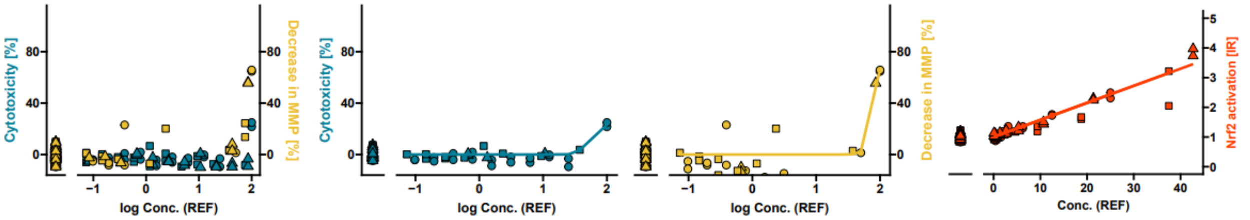

(i) S74_20190606

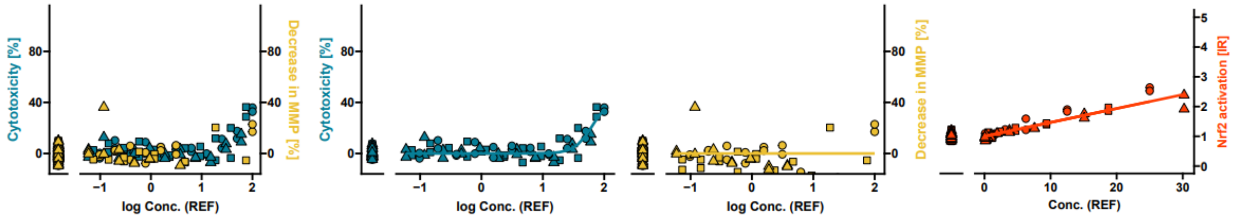

(j) S76_20190511

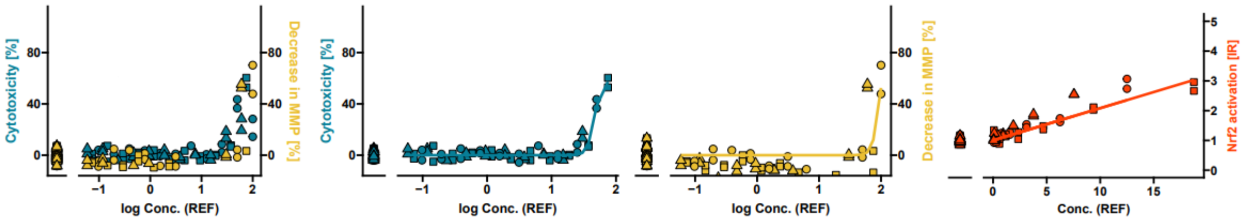

Figure S7. Continued. Concentration-response curves of surface water and wastewater treatment plant (WWTP) effluent for cytotoxicity, MMP disruption and oxidative stress response in AREc32 cells.

(k) S79_20190516

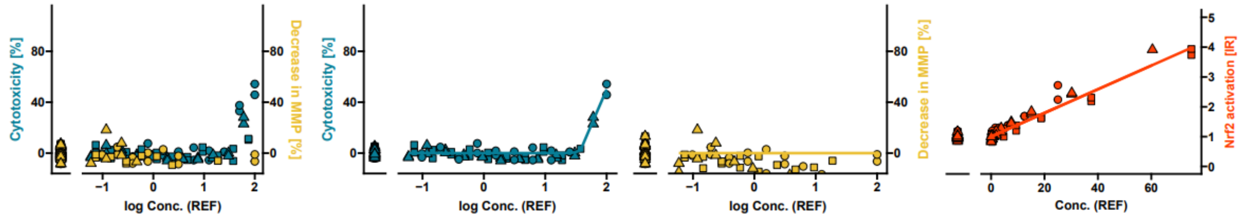

(l) S81_20190520

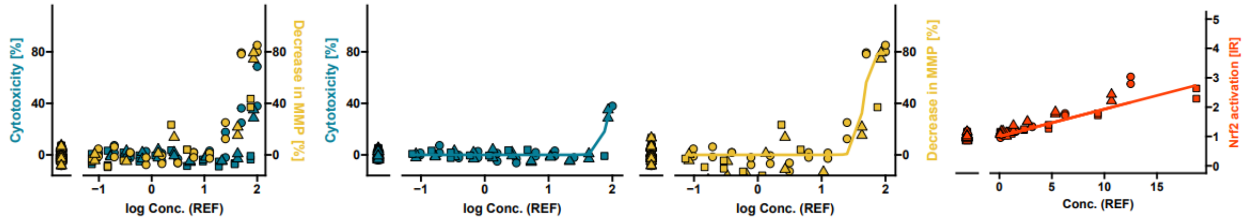

(m) S83_20190520

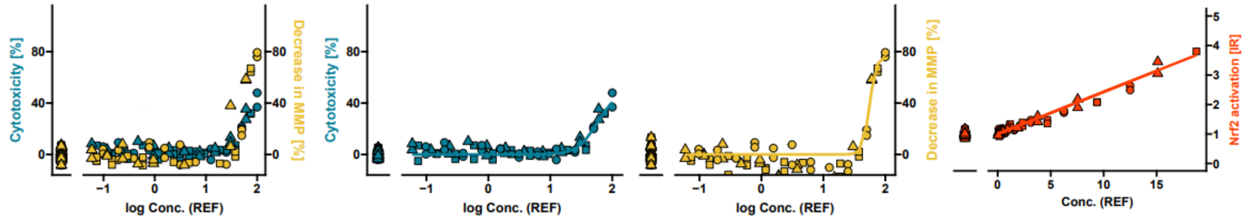

(n) S83_20190611

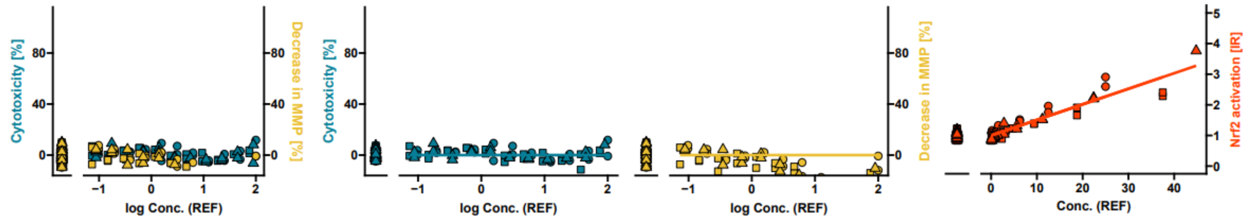

(o) S92_20190509

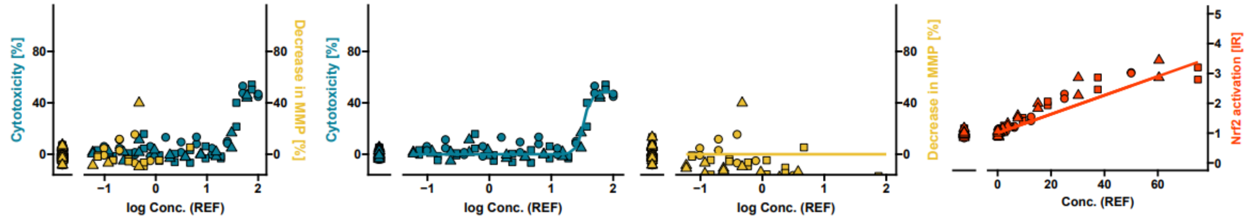

Figure S7. Continued. Concentration-response curves of surface water and wastewater treatment plant (WWTP) effluent for cytotoxicity, MMP disruption and oxidative stress response in AREc32 cells.

(p) S92_20190611

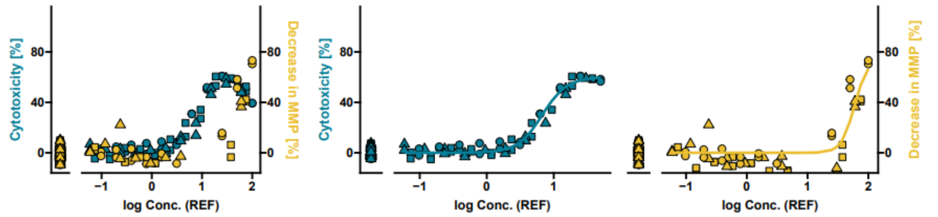

(q) S95_20190611

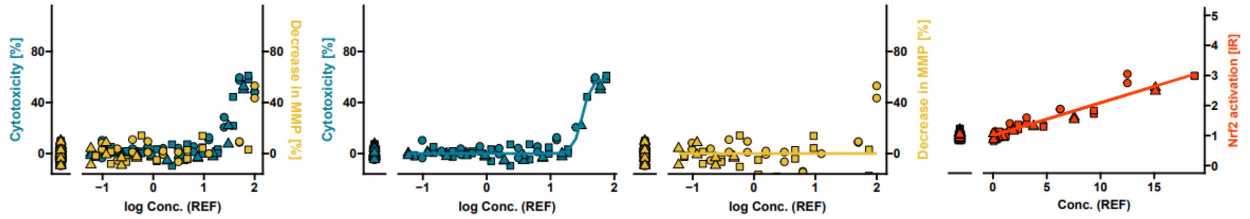

(r) S98_20190511

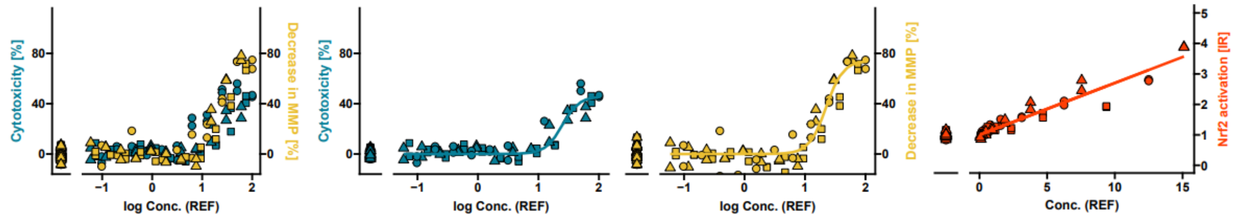

(s) S98_20190528

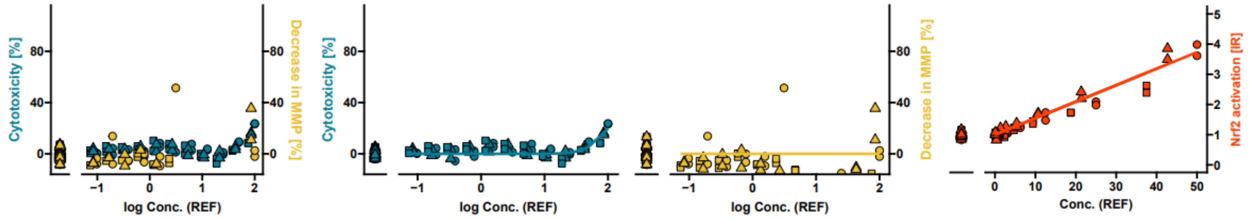

(t) S101_20190520

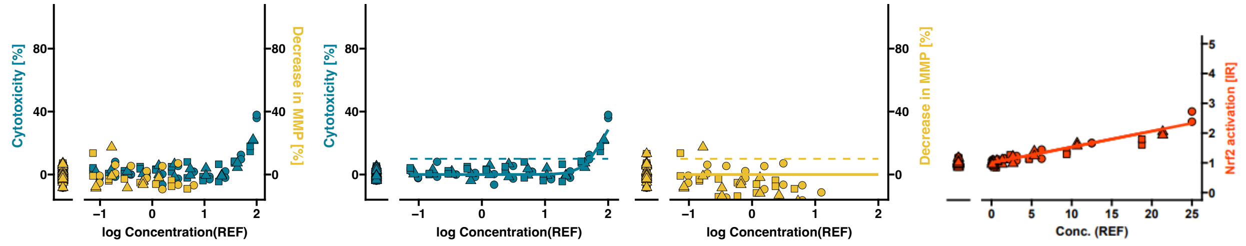

Figure S7. Continued. Concentration-response curves of surface water and wastewater treatment plant (WWTP) effluent for cytotoxicity, MMP disruption and oxidative stress response in AREc32 cells.

Wastewater treatment plant (WWTP) effluent

(a) EU001

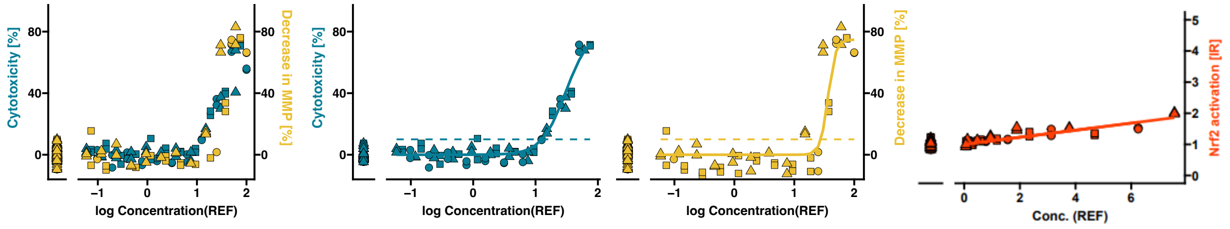

(b) EU003

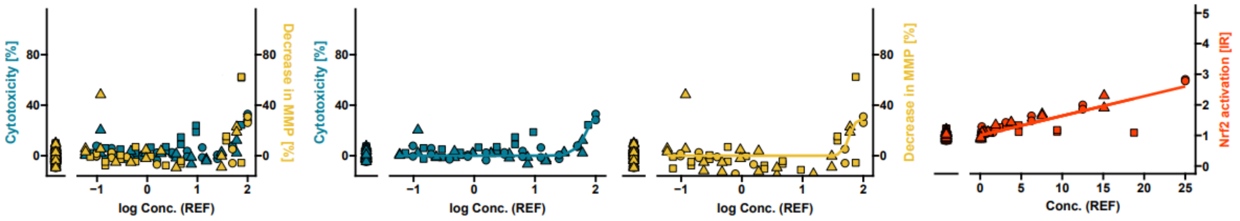

(c) EU009

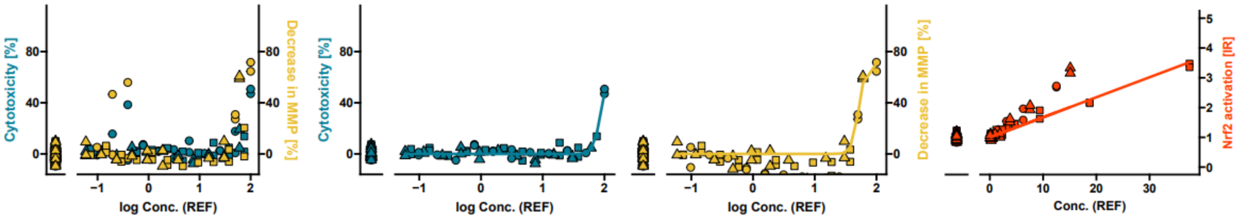

(d) EU011

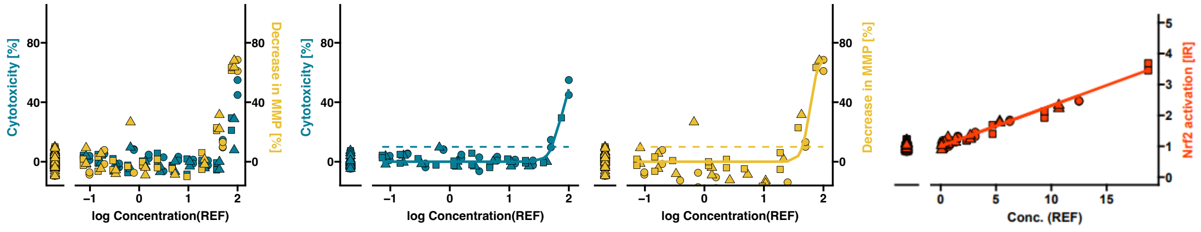

(e) EU017

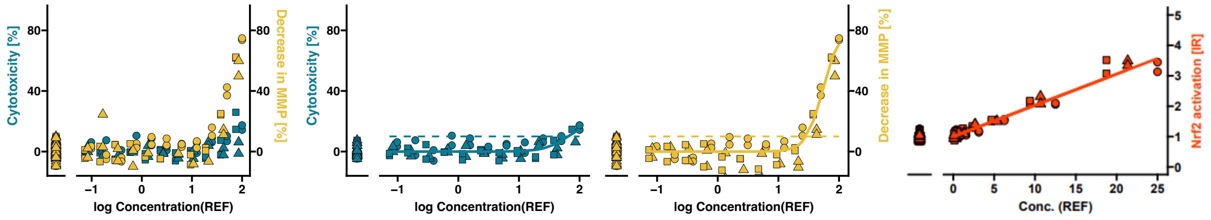

Figure S7. Continued. Concentration-response curves of surface water and wastewater treatment plant (WWTP) effluent for cytotoxicity, MMP disruption and oxidative stress response in AREc32 cells.

(f) EU018

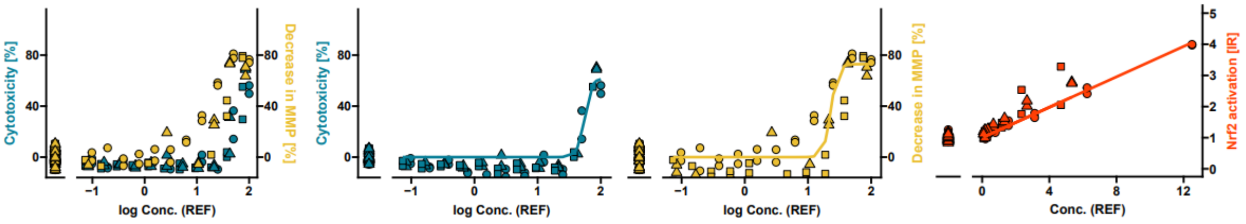

(g) EU019

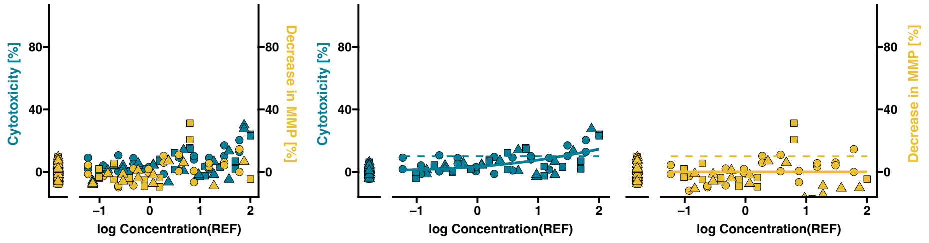

(h) EU022

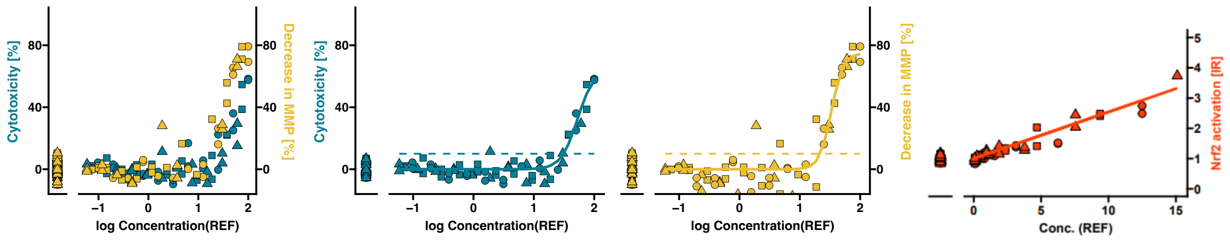

(i) EU023

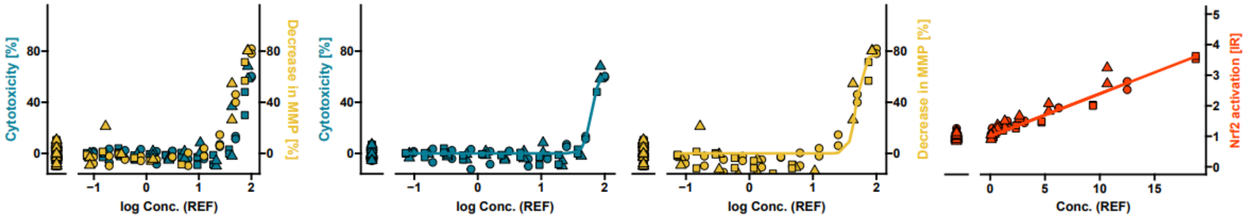

(j) EU032

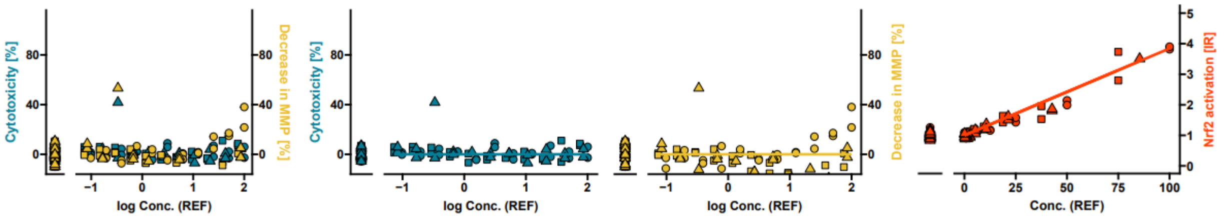

Figure S7. Continued. Concentration-response curves of surface water and wastewater treatment plant (WWTP) effluent for cytotoxicity, MMP disruption and oxidative stress response in AREc32 cells.

(k) EU102

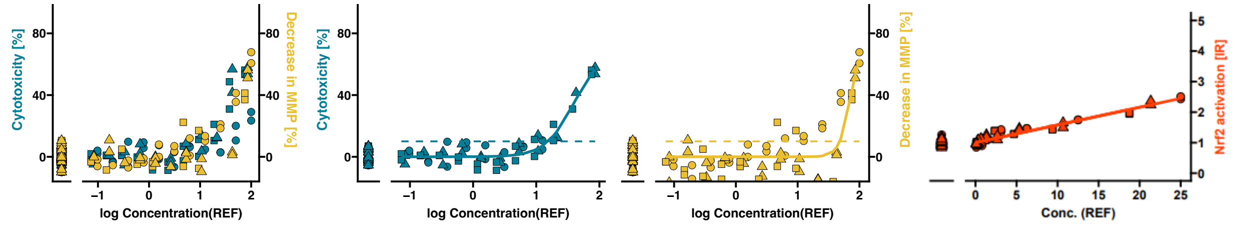

(l) EU120

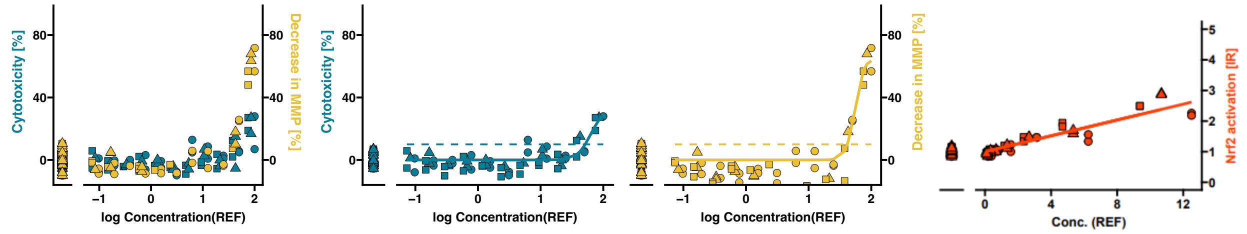

(m) EU121

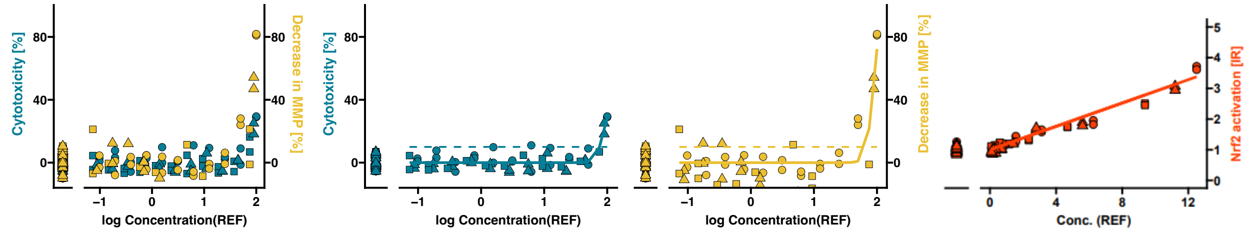

(n) EU122

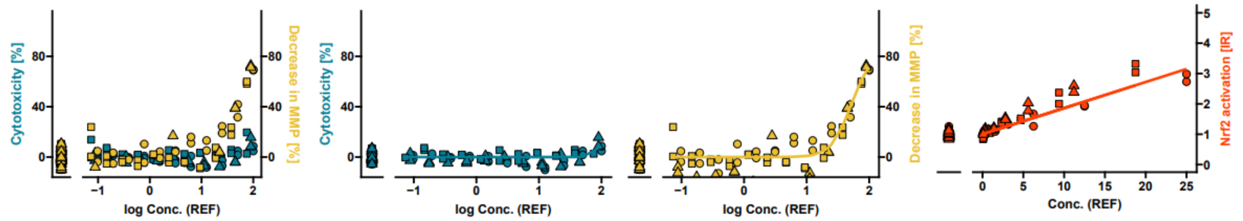

(o) EU123

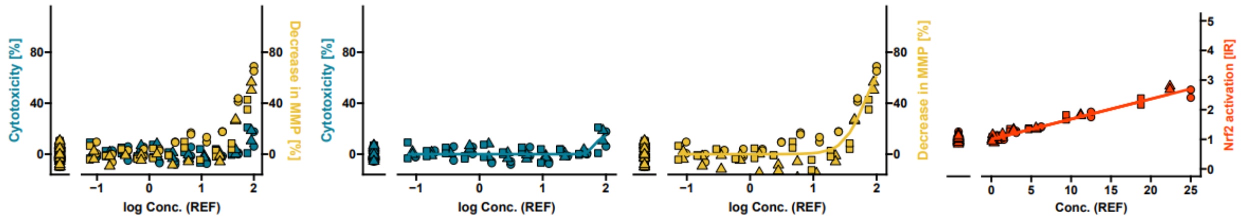

Figure S7. Continued. Concentration-response curves of surface water and wastewater treatment plant (WWTP) effluent for cytotoxicity, MMP disruption and oxidative stress response in AREc32 cells.

(p) EU124

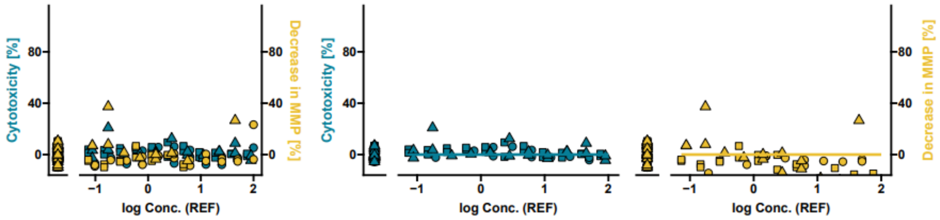

(q) EU128

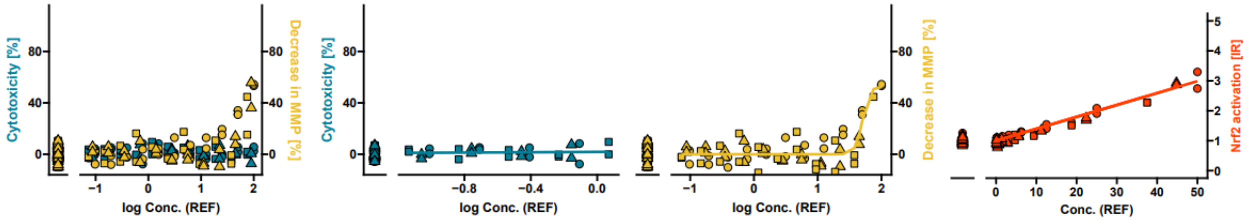

(r) EU129

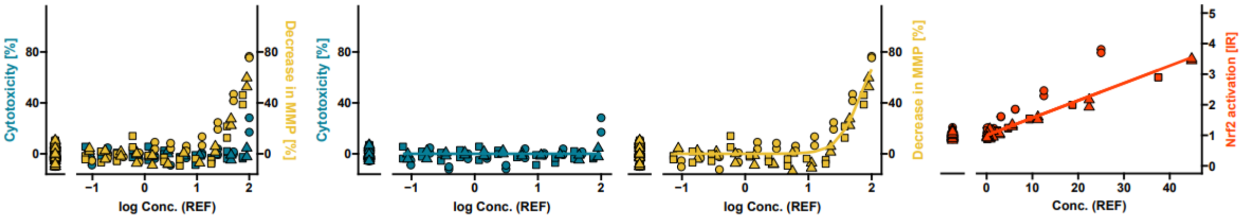

(s) EU130

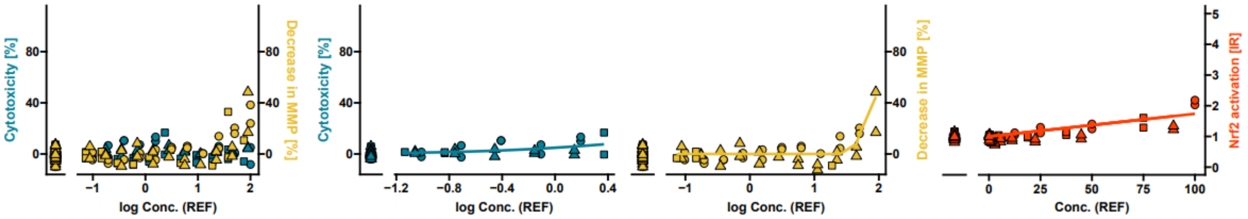

(t) EU131

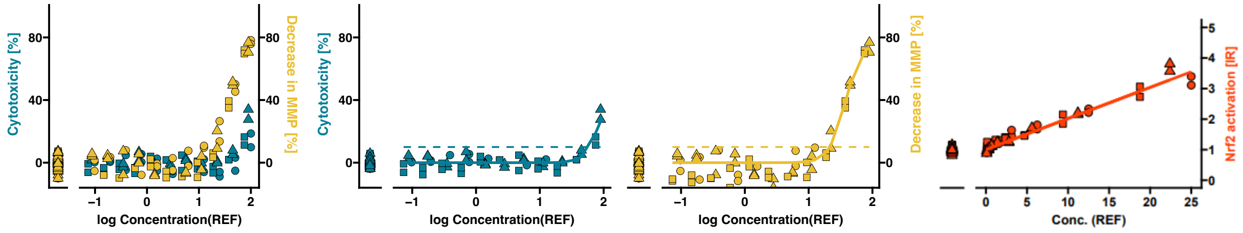

Figure S7. Continued. Concentration-response curves of surface water and wastewater treatment plant (WWTP) effluent for cytotoxicity, MMP disruption and oxidative stress response in AREc32 cells.

Table S3. Effect concentrations of surface water and wastewater treatment plant (WWTP) effluent for cytotoxicity, MMP disruption and oxidative stress response in AREc32 cells – see Supporting Information excel file.

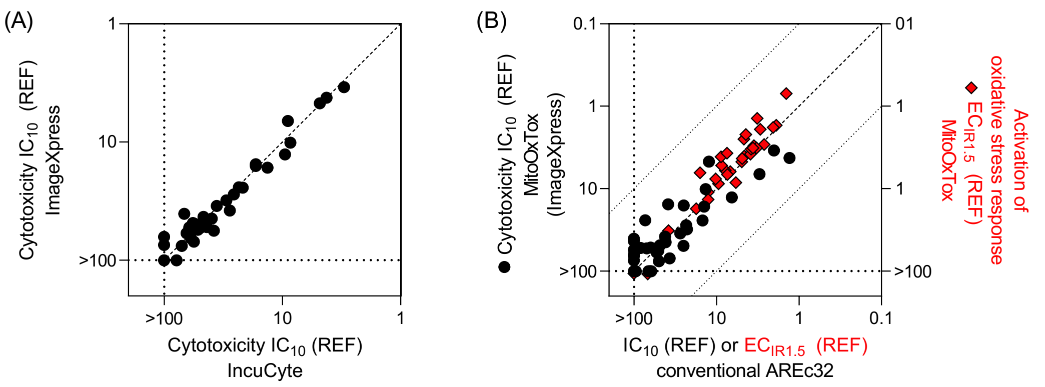

Figure S8. (A) Comparison of cytotoxicity measured with ImageXpress and IncuCyte for water extracts in AREc32 cells. (B) Comparison of the IC_{10} for cytotoxicity and $EC_{IR1.5}$ for the activation of oxidative stress response in AREc32 cells using the conventional setup[1] and the new multiplexed MitoOxTox assay.

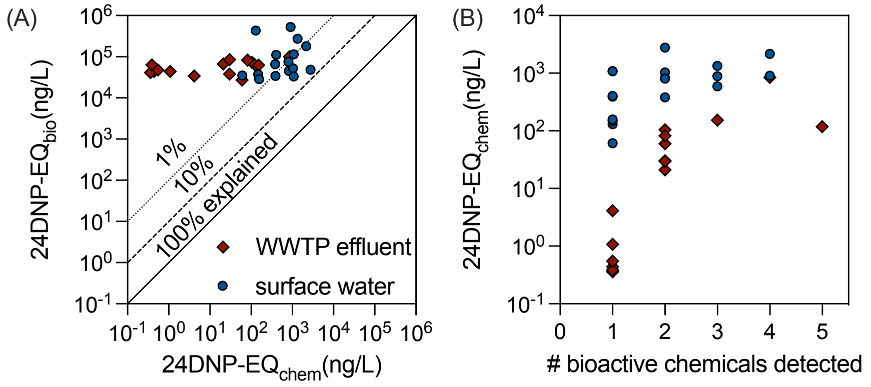

Figure S9. (A) Comparison of 24DNP-EQ_{bio} and 24DNP-EQ_{chem} for the MMP endpoint for surface water samples (blue circles) and WWTP effluents (red diamonds). (B) Relationship between number (#) of chemicals detected and 24DNP-EQ_{chem}.

Table S4. Iceberg modelling for MMP disruption of surface water and waste water treatment plant (WWTP) effluent – see Supporting Information excel file.

Text S1. Simplification of experimental procedure.

To simplify the procedure of multiplexed assay, we aimed to use single type of assay plate over the whole procedure for measuring three endpoints. Originally, a black wall plate is recommended for fluorescence measurement to reduce background and crosstalk of fluorescent signal. Conversely, use of a white wall plate is recommended when measuring luminescence, which is oxidative stress response measurement in our case, due to maximal light output from its reflective properties.

The AREc32 cells were seeded in a white wall/clear bottom 384-well plate (Greiner, 781944) and the assay plate was processed with the same procedure for chemical exposure and image acquisition as described in the main manuscript. In the luciferase assay, the cells were lysed, and the substrate buffer was directly added to the original plate without transferring step. All other steps were identical. For this verification, 20 chemicals/samples were selected considering their activity in the original procedure.

While we observed that using a black plate for luminescence measurement critically affected measured values, use of a white wall plate seems to cause minor changes (data not shown). Hence, we compared IC_{10}, EC_{10}, and $EC_{IR1.5}$ in Table S2 and S3 which were measured with original procedure (where the most suitable plate type is used for fluorescence and luminescence measurement) with those from the simplified procedure where only a white wall plate was used. Overall, all IC_{10}, EC_{10}, and $EC_{IR1.5}$ were similar between original and simplified procedure considering all three effect concentrations having difference within a factor of magnitude (Figure S10). Especially, in MMP measurement where crosstalk might occur, we still observed similar level of EC_{10} values between the two procedures (Figure S10B). In case of fenazaquin, EC_{10} differed with 9-fold difference since its CRC did not reach 0% level properly at the lowest tested concentration and hence EC_{10} was not determined solidly enough from both procedures. This simplification of the assay procedure is advantageous considering use of 50% fewer plates and time efficiency by skipping the transfer procedure.

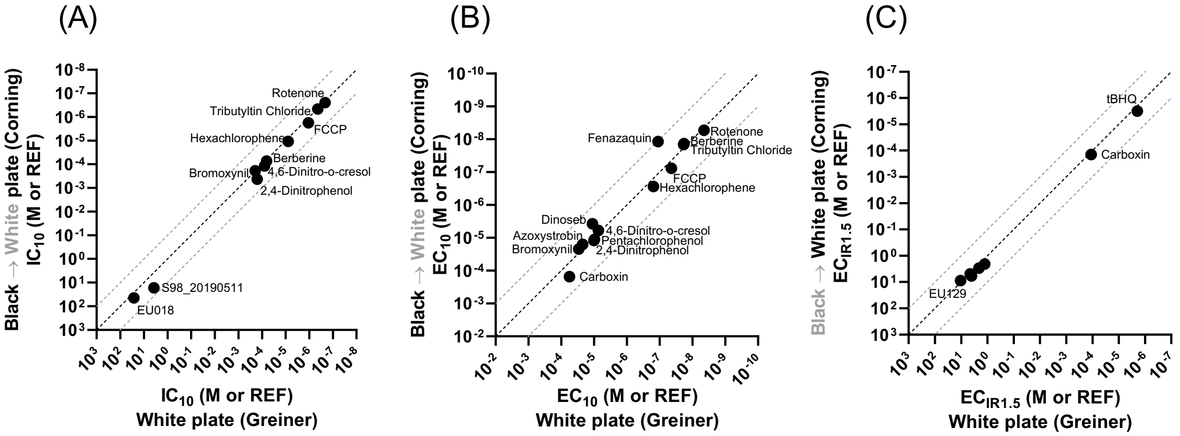

Figure S10. Comparison of (A) IC_{10}, (B) EC_{10}, and (C) $EC_{IR1.5}$ between experiments using Greiner plates with white wall/clear bottom for all three endpoints and different plates for detection of the different endpoints (black wall/clear bottom plate for cell cytotoxicity and MMP measurement from Corning and white wall/clear bottom plate for oxidative stress response assay from Corning).

References

(1) Lee, J.; Schlichting, R.; König, M.; Scholz, S.; Krauss, M.; Escher, B. I. Monitoring mixture effects of neurotoxicants in surface water and wastewater treatment plant effluents with neurite outgrowth inhibition in sh-sy5y cells. *Environmental AU* **2022**. DOI: 10.1021/acsenvironau.2c00026.
